# Biophotonics point-of-care diagnostics in low-resource settings: a South African perspective

**DOI:** 10.1117/1.JBO.31.11.113506

**Published:** 2026-09-28

**Authors:** Patience Mthunzi-Kufa, Morongwa Mary Mathipa, Tendai Makwikwi, Mellisa Brenda Sagandira, Moses Wabwile Juma, Degratious Kgoale, Nkgaphe Tsebesebe

**Affiliations:** a University of South Africa, College of Science, Engineering and Technology, School of Science, Florida, South Africa; b University of South Africa, College of Agriculture and Environmental Sciences, Department of Environmental Sciences, Florida, South Africa; c Great Zimbabwe University, School of Medicine and Health Sciences, Department of Biomedical Sciences, Masvingo, Zimbabwe; d Midlands State University, Chemical Sciences Department, Senga Gweru, Zimbabwe; e Kibabii University, Department of Science, Technology and Engineering, Bungoma, Kenya

**Keywords:** point-of-care diagnosis, low-resource settings, communicable diseases, photonics devices, biophotonics

## Abstract

**Significance:**

Point-of-care (POC) diagnostics based on photonics and biophotonics technologies hold transformative potential for healthcare delivery in peri-urban and underdeveloped settings, combining optical sensitivity with portability, rapid readout, and minimal consumable requirements.

**Aim:**

We aim to synthesize design considerations and recent advances that align photonics-based POC devices with the World Health Organization’s ASSURED/REASSURED framework, emphasizing affordability, sensitivity, ease of use, rapidity, robustness, equipment-free operation where possible, deliverability, and the added priorities of real-time connectivity and environmental sustainability.

**Approach:**

We examine implementation evidence from sub-Saharan Africa, highlighting gaps in field validation, quality assurance, and sustainability identified by recent scoping reviews, as well as the persistent need for context-adapted usability and training strategies. Advances covered include miniaturized optical biosensors (fluorescence, Raman, and photothermal modalities), smartphone-integrated microscopy and spectroscopy, low-cost LASER/LED illumination systems, and multiplexed lateral-flow/optical hybrids, all tailored for low-resource constraints.

**Results:**

We discuss how digitization and connected diagnostics (including data standards, mobile health integration, and secure cloud reporting) amplify clinical impact, enable surveillance, and support task-shifting in primary care, aligning with South Africa’s National Digital Health Strategy. We also survey regional innovation capacity, with examples of locally focused development and prototyping that demonstrate pathways to regional manufacturing and deployment.

**Conclusions:**

We conclude with recommendations for regulatory-science partnerships, human-centred design, interoperable digital pipelines, and investment in field-based validation and manufacturing to translate photonics innovations into resilient, equitable POC solutions across South Africa and the wider sub-Saharan context.

## 1 Introduction

In developing countries, patients in peri-urban and rural communities face multifaceted barriers to timely diagnosis, including limited laboratory infrastructure, shortages of trained laboratory personnel, and fragile specimen transport, which frequently results in long turnaround times or lost results that delay clinical decision-making.^1^ Long travel distances, unreliable transport, and the direct costs of repeat clinic visits further discourage care-seeking and follow-up, so many individuals present late or fail to receive confirmatory testing.^2^ Health-system bottlenecks, such as specimen losses throughout the pathology value chain and backlogs in specialized services (e.g., histopathology), exacerbate these access problems, leading to missed or delayed diagnoses.^3^ The combined effect is evident across high-burden conditions, for example, tuberculosis (TB), HIV-related opportunistic disease, and cancers are frequently diagnosed at more advanced stages in resource-constrained districts, increasing morbidity, treatment complexity, and onward transmission.^3^ These structural and logistical gaps underscore the urgent need for context-adapted POC diagnostics, locally appropriate workflows, and strengthened digital as well as specimen-tracking systems in areas where national actors and research organizations are actively developing tailored POC solutions.^4^ The South African healthcare landscape presents a complex interplay of significant opportunities and structural constraints that directly shape the deployment and impact of POC diagnostics, including emerging biophotonics-enabled modalities. As a middle-income nation with profound disparities across geography and socioeconomic strata, South Africa’s public health system is characterized by centralized laboratory networks that struggle to meet diagnostic demand in rural and underserved areas, resulting in prolonged turnaround times and diagnostic delays that compromise patient management and continuity of care. Qualitative investigations in primary healthcare clinics have revealed that while basic POC tests (e.g., HIV, glucose, pregnancy) are available, challenges in scaling up services are compounded by staff training gaps, unreliable quality management, and supply chain limitations, all of which undermine test reliability and sustainability in rural settings.^5^ Concurrently, stakeholders involved in digital-linked POC diagnostic implementations highlight limited connectivity, intermittent electricity supply due to load shedding, and restricted access to enabling technologies as significant barriers to widespread adoption, even as ease of use and system support are identified as critical enablers.^6^ These constraints are juxtaposed against strong policy momentum, such as plans to integrate POC diagnostics within South Africa’s National Health Insurance framework to decentralize care and improve equity and recognition of the potential for rapid, near-patient testing to accelerate clinical decision-making and reduce healthcare costs.^5^ In this context, advancing POC diagnostics, particularly optical biosensors and other biophotonics-based tools, demands not only technological innovation but also investment in infrastructure, workforce training, and quality assurance systems to ensure their effective, equitable implementation across the country’s diverse health settings.^5^

Photonics and biophotonics hold transformative promises in addressing diagnostic inequity by enabling highly sensitive, rapid, and portable modalities that can be deployed at or near the POC, particularly in underserved and resource-limited settings where traditional laboratory infrastructure is scarce. For example, optical biosensors, which encompass fluorescence, surface plasmon resonance, Raman, and other spectroscopy-based techniques, offer label-free, real-time detection of disease biomarkers with minimal sample preparation and infrastructure demands, making them ideal candidates for decentralized diagnostics.^7^

Integrated optical biosensors, often coupled with microfluidics and miniaturized detection platforms, have demonstrated the potential to achieve laboratory-comparable sensitivity and specificity in a compact format suitable for field use.^8^ Such advances support the World Health Organization’s ASSURED criteria for low-resource diagnostics (affordable, sensitive, specific, user-friendly, robust, equipment-free, and deliverable to end-users). Moreover, novel portable optical imaging technologies have been demonstrated to enhance the efficacy of early cancer detection and screening in low-resource environments by enabling noninvasive, wide-field, and high-resolution visualization of pathological changes, without reliance on extensive infrastructure or specialized interpretation.^9^ The integration of artificial intelligence (AI) and smartphone connectivity further enhances data interpretation and real-time decision support, thereby reducing dependence on highly trained personnel and bridging gaps in diagnostic capacity. Collectively, these photonics-based innovations hold considerable potential to reduce disparities in access to timely and accurate diagnostics, which is essential for equitable healthcare delivery globally.

This review examines the emerging role of biophotonics and photonics-enabled POC diagnostics in addressing diagnostic inequity, with a particular focus on low-resource and underserved settings such as those found in South Africa and comparable regions globally. The scope encompasses both foundational optical sensing and imaging principles, as well as recent translational advances in portable, low-cost, and digitally augmented photonics platforms designed for decentralized clinical use. The primary objectives are threefold: (1) to contextualize diagnostic inequity by analyzing the health-system, infrastructural, and socio-economic barriers that constrain access to traditional laboratory diagnostics; (2) to synthesize current progress in photonics POC technologies, including optical biosensors, spectroscopy-based assays, miniaturized imaging systems and smartphone-integrated tools and evaluate their readiness, performance, and suitability for low-resource deployment; and (3) to outline the translational challenges, regulatory considerations, and implementation pathways required to ensure equitable adoption and long-term sustainability of these technologies. The review is structured to first provide an overview of diagnostic inequity and healthcare constraints, followed by a detailed examination of biophotonics principles relevant to POC applications. Subsequent sections analyze key technology categories, highlight case studies demonstrating field translation, and assess barriers to scale-up such as cost, power reliability, quality management, and workforce training. The final sections propose strategic recommendations for research, policy, and capacity development to accelerate the integration of biophotonics into equitable global diagnostic ecosystems.

## 2 Fundamentals of Biophotonics Diagnostics

### 2.1 Light–Tissue Interaction Principles Relevant to POC Applications

Optical approaches provide noninvasive, rapid and low-cost sensing mechanisms that could be ideal for point of care diagnostic applications. The performance of any optical technique will depend on how light propagates and subsequently interacts with tissues and the emerging chemical and structural signals.^10^ Light of sufficient energy may interact with tissues through absorption, scattering, reflection, or fluorescence, such that if the interaction is noninvasive, the molecular information of the tissues can be easily discerned.^11^ Light absorption determines the extent to which light can penetrate a given tissue with the absorption properties depending strongly on the wavelength and is important in diagnostics and the therapy of abnormal tissue conditions.^12^ Krasnikov et al.^12^ conducted a mini-review on the fundamentals of light–matter interaction describing how light is used in imaging, diagnosis, therapy and surgery. Although noting that the use of modern light in medicine begun in the nineteenth century, Krasnikov et al.^12^ noted rapid improvements in understanding the physical nature of light and the fundamental light–matter interactions. For instance, phototherapy used the ultraviolet (UV)-induced treatment of *Lupus vulgaris* with the development of lasers leading to other medical avenues.^10^ On the contrary, the scattering process can alter the path taken by photons, and the spectrum of the scattered light, eventually being mapped for diagnosis and tissue imaging.^10^ For instance, Steelman et al.^13^ highlighted some of the biomedical light scattering techniques, including elastic light scattering, diffuse reflectance spectroscopy, and four-dimensional elastic light scattering fingerprinting for early colon carcinogenesis.^14^ The techniques form part of the current state of photonic technologies that utilize light scattering for disease detection since they measure the spectral, angular, and phase properties of the scattered field. Figure 1 illustrates the light-tissue interaction mechanism for biological tissues.

**Fig. 1:**
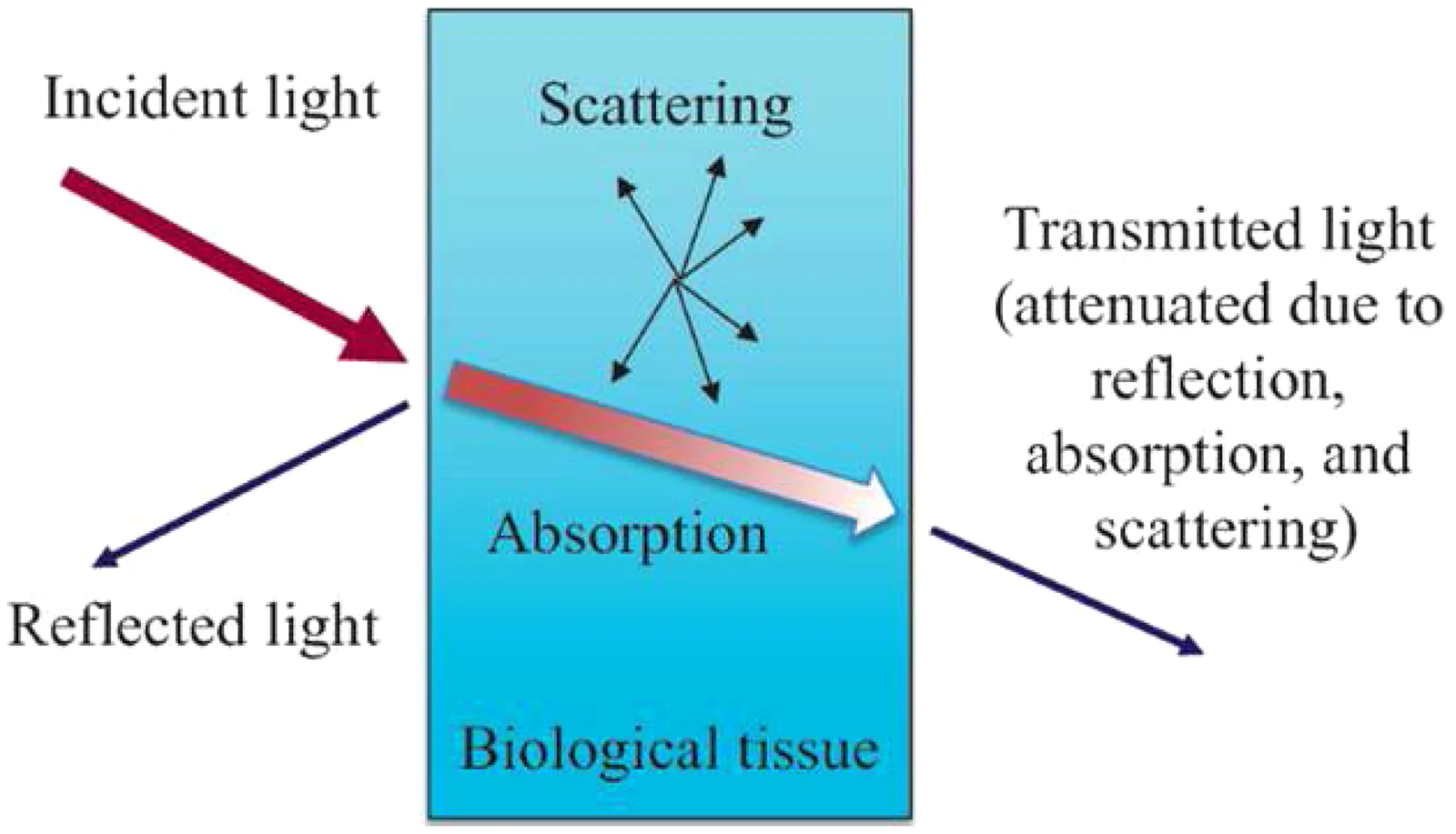
Scattering and absorption mechanism as light interacts with a biological tissue.^15^

As noted in Fig. 1, incident light may be reflected, transmitted, or absorbed through various interaction mechanisms, providing valuable insights into the optical properties of biological tissue. The absorption-based spectroscopy relies on the Beer–Lambert law which states that the concentration of the analyte is directly proportional to the light absorbance as described in Eq. (1).

A=ϵ×C×L, (1)

where ϵ is the molar extinction coefficient describing how strong the tissues absorb light, L is the optical path, and C is the analyte concentration. Soliman et al.^16^ noted that by measuring the absorbed light, it is possible for an optical-based device to quantify disease markers, such as specific proteins or the viral loads, providing a rapid diagnostic approach.

### 2.2 Major Optical Modalities for Biomedical Diagnostics

#### 2.2.1 Fluorescence and raman spectroscopy

Fluorescence and Raman spectroscopy are two optical techniques often used in biomedical diagnostics. Fluorescence spectroscopy measures light emitted by molecules after excitation at shorter wavelengths and characterizes the unique emission spectra that provide information about a material.^17^ However, despite the high signal intensity and broad emission peaks, fluorescence suffers from spectral peak overlap, which limits its use in biomedical diagnostics. Wang et al.^18^ noted that while fluorescence spectroscopy is an emerging technique that can be employed in diseases such as early cancer diagnostics, there is a need for more clinical trials to be carried out to guarantee the validity of the technique.

Conventional Raman spectroscopy, or its modified version, surface-enhanced Raman spectroscopy (SERS), often addresses some of the challenges of fluorescence, particularly in early disease diagnostics. Conventional Raman spectroscopy is both rapid and noninvasive, preserving sample integrity, and can act as a fingerprint of biological materials such as tissues.^19^ Raman spectroscopy relies on the Raman effect which involves the inelastic scattering of monochromatic light such as a laser by the vibrating molecules with most of the light scattering elastically without a change in energy while a fraction of the light interacts with the molecular bonds causing the scattered photons to gain or lose energy. This change in energy corresponds to the specific vibrational mode of the molecule creating its molecular fingerprint.^20^

Detection of trace and ultra-trace biomolecules in POC, such as detecting cancer biomarkers, has also been achieved by using SERS. According to Patel et al.,^21^ disease biomarkers can be detected sensitively, enabling early disease diagnostics. The capability of SERS is derived from modifying the substrate surface using nanomaterials such as gold or silver, achieving an enhanced signal, for ultra-trace biomedical diagnostics.^22^ Although absorbance, scattering, and fluorescence are the most common optical detection modalities for POC diagnostics, other methods such as chemiluminescence and bioluminescence are also used, especially in immunoassay formats where enzyme-labeled probes generate light signals without external excitation.

#### 2.2.2 Optical coherence tomography and scattering-based methods

Like Raman spectroscopy, optical coherence tomography (OCT) is a noninvasive imaging technique that uses infrared radiations for high-resolution 3D imaging for better visualization of body tissues.^23^ New frontiers in OCT technology have enabled the imaging of nontransparent tissues, making it useful in medical diagnostics.^24^ Using light waves, OCT can measure the thickness of the retinal layer, making it possible to have a 3D view of the internal structure of the eye.^25^ Fan et al.^26^ noted that by integrating laser scanning capabilities, it is possible to use OCT for three-dimensional reconstruction of tissue structures. In addition, most recently developed OCT systems operate in the NIR range, which allows lower scattering coefficients and higher absorption for small layer penetration in biological tissues.^27^

Besides OCT, other scattering-based approaches are employed in biomedical diagnostics. As previously discussed, Raman spectroscopy is an example of an elastic scattering method whose spectral data can act as biomarkers for characterizing morphological properties of tissues.^28^ Mourant et al.^28^ developed an optical biopsy system that is based on elastic scatter spectroscopy, where light is delivered to the tissues using optical fibers. The approach was tested on bladder cancer, the diagnosis of dysplasia in the oesophagus, and in detecting adenomatous polyps, with the authors reporting reliable results. Other scattering techniques used in biomedical applications include diffuse reflectance spectroscopy employed in the diagnosis of skin cancer and peripheral lung tumors,^29^ and light scattering spectroscopy.^30^ The advantage of scattering techniques is being nonionizing and can provide real-time, noninvasive feedback by analyzing how light scatters at different angles and wavelengths to reveal morphological and biochemical changes indicative of disease.^31^

#### 2.2.3 Diffuse optical imaging and near infrared spectroscopy

Diffuse optical imaging (DOI), diffuse optical tomography, and near-infrared spectroscopy (NIRS) are noninvasive techniques that create images and measure the physiological properties of biological tissues.^32^ NIRS utilizes light in the NIR region (∼700 to 2500 nm spectral window), offering a noninvasive approach to analyzing various molecules.^33^ In tissue diagnostics, near-infrared light is absorbed and scattered by tissue to create a 3D picture of its optical properties, which can reveal functional information about the tissue. DOI can assess parameters such as blood oxygenation, blood flow, and tissue composition, making it applicable in breast cancer imaging, tissue composition assessment, and blood flow evaluation, among other applications.^34^ DOI addresses the limitations of conventional NIR while also offering potential for diagnostic optical imaging.^35^

#### 2.2.4 Photonic and plasmonic biosensors

Photonic and plasmonic biosensors are advanced optical tools that utilize light-based phenomena to sensitively and rapidly identify clinical biomarkers.^36^ Plasmonic biosensing has recently been used to achieve rapid, real-time, and label-free analysis, especially for small molecules at low concentrations, producing compact devices that achieve POC diagnostics.^36^ The sensing principle of photonic and plasmonic biosensors involves measuring shifts in resonance peaks resulting from the binding of nanostructures to the target analyte. When nanomaterials are used as binding sites, the localized surface plasmon resonance (LSPR) phenomenon is employed, allowing single-molecule detection by utilizing changes in optical properties.^37^ Juma et al.^38^ demonstrated the use of silver nanoparticles for LSPR-based sensing of ultra-trace trenbolone acetate dopants.

Plasmonic and photonic biosensors can be used in POC diagnostics by creating portable, sensitive, and rapid detection systems for disease biomarkers. According to Butt,^39^ plasmonic sensors utilize the interaction of light with nanomaterials, such as gold and silver, for the label-free detection of biomolecules through surface plasmon resonance (SPR) or LSPR approach. For SPR, incident light interacts with delocalized electrons at the metal-dielectric interface to launch surface plasmon waves. This is highly sensitive to bulk refractive index changes, making it the standard for monitoring biomolecular interactions. On the contrary, LSPR uses nanoscale structures (such as nanoparticles or nanorods). Instead of propagating along a film, the plasmons are localized to the nanoparticle, allowing for highly miniaturized and cost-effective sensors and POC diagnostic tools as reported by Jin et al.^40^

#### 2.2.5 Compact microscopy and smartphone-based imaging

Compact microscopy and smartphone-based imaging platforms are transforming biomedical diagnostics by providing portable, cost-effective, and highly connected POC solutions.^41^ Compact microscopes, such as lensless holographic microscopes with detection schemes, have recently been reported by Hernández-Neuta et al.^42^ as effective approaches for medical image processing. The advancement of micro-optics, solid-state lasers, and optical fibers has been a game-changer in the development of biomedical devices either as miniaturized microscope architectures or as smartphone applications.^42^

Smartphone-based approaches have emerged, motivated by the continued adoption of smartphone devices in everyday life, making it possible for them to be integrated in solving sensing and diagnostic needs.^43^ Key capabilities such as being easily portable and higher functionality levels have provided seamless capability of integration smartphones in developing diagnostic devices.^44^ According to Kumar et al.,^45^ smartphone-based imaging provides promising alternatives in evidence-based care, especially in resource-limited settings. Although smartphones are not often designed for medical diagnostics, they can be adapted for such purposes using modified hardware that enable microscopic imaging and interface with diagnostic tests. As technology improves, including the adoption of the 5G network, there will be better POC diagnostics, simplifying healthcare workflows and reducing patient waiting time.^46^

### 2.3 Comparative Advantages for Low-Resource Deployment

Point of care diagnostics provide significant advantages in low-resource settings (LREs) by allowing immediate clinical decision-making. Such diagnostic processes reduce the need for centralized laboratory infrastructure, empowered primary care providers and minimizing turn-around times per-patient especially for conditions that are time sensitive.^46^ According to Elendu et al.,^46^ improved access to care in remote areas, faster and more efficient diagnostic processes, cost-effectiveness, and ease of use by minimally trained personnel are feasible in point-of-care diagnostic processes. Existing diagnostic instrumentation typically requires sophisticated infrastructure that is hardly available in resource-limited settings like in the case of developing countries.^47^ Consequently, innovative approaches, including low-resource deployment, can enhance the deployment of alternative diagnostic approaches that guarantee quality healthcare delivery.^47^

## 3 Point-of-Care Design Considerations in Low-Resource Environments

### 3.1 The ASSURED and REASSURED Criteria for Diagnostic Tools

The ASSURED framework—Affordable, Sensitive, Specific, User friendly, Rapid and robust, Equipment free, and Deliverable established by World Health Organization (WHO)—has served as the foundational model for POC global health diagnostics in the last 20 years.^48^ This approach has been instrumental in aligning engineering efforts with the key challenges of LREs, focusing not only on analytical performance but also on pragmatic concerns such as usability, cost, and distribution.^49^ Nonetheless, the swift progress of digital health and a deeper understanding of health system integration have highlighted limitations in this original model, prompting its evolution into the more comprehensive REASSURED model.^50^

The elemental ASSURED principles remain critically pertinent.^49^ Affordability extends beyond the initial device cost to encompass the total cost of ownership, including maintenance, consumables, and operational overhead, which must be sustainable within the constrained budgets of public health systems in resource low-resource settings. Sensitivity and specificity are indispensable, in tests administered in low-resource settings should not compromise accuracy, as false positives and negatives can result in severe individual and public health implications, ultimately eroding trust in the health system. The tenets of user-friendliness, rapidity, and robustness are fundamentally interconnected, requiring interfaces that minimize cognitive load and manual steps for nonspecialist users, while withstanding environmental stressors such as humidity, dust, and fluctuating temperatures. The equipment-free ideal, although hardly wholly achievable for complex assays, exhorts innovation toward miniaturization and simplification, and deliverability warrants that the diagnostic can survive supply chain logistics and reliably reach its destination.

The transition to REASSURED criteria encompasses two transformative additions that consider the realities of 21st-century healthcare.^51^^–^^53^

1. Real-time connectivity. The inclusion of the first ‘R’ marks a sea change from detached diagnostic devices to interlinked nodes in a digital health ecosystem. This potential for real-time or near-real-time data transmission is a game-changer for syndromic surveillance, enabling rapid detection and response to outbreaks. When managing chronic diseases such as hypertension or HIV, it enables longitudinal patient monitoring and adherence assessment. Connectivity revolutionizes a diagnostic result from a discrete event into a valuable input that can inform health strategy. The selection of a suitable connectivity protocol (i.e., Bluetooth, satellite, or GSM) must be determined by the telecommunication infrastructure of the target environment to warrant functionality.
2. Ease of specimen collection. This benchmark recognizes that the patient experience and the biosafety of sample acquisition are key factors of successful implementation. Invasive techniques such as venipuncture necessitate skilled phlebotomists, dedicated equipment, and pose biohazard risks. The REASSURED framework, therefore, endorses minimally invasive sampling techniques, such as finger-stick blood, saliva, or urine, which reduce the barrier to testing and thereby increase patient acceptability, simplifying the workflow for community health workers. This perfectly aligns with the critical need of designing diagnostic pathways that are not only clinically efficient but also operationally and culturally feasible.

The REASSURED criteria, thus, represent a universal, system-level view of diagnostic impact. This framework challenges developers to design tools that are not simply systematically sound but also cost-effective, digitally integrated, and patient centred.^54^ Nonetheless, it is essential to acknowledge that REASSURED is a framework, not a universal solution. Its successful employment is dependent upon parallel developments in regulatory harmonization across low- and medium-income countries (LMICs), the development of sustainable market models, and a multifaceted, localized appreciation of the cultural and operational frameworks in which these transformative diagnostics will be deployed.

### 3.2 Engineering Design for Portability, Robustness, and Ease of Use

The use of diagnostic technologies in LREs presents a significant engineering challenge that exceeds traditional performance metrics. Success in this domain is not simply a function of analytical sensitivity or specificity but is essentially dominated by a device’s resilience to external difficulties and its key operational simplicity. Effective design for LREs needs an ultimate shift from ‘glocalization’ to frugal innovation.^55^ Instead of simplifying complex lab instruments inefficiently, we must be guided by a first-principles approach to build robust, affordable, and high-quality solutions that operate within severe limitations. This design philosophy is manifested through various core engineering pillars, including robustness, portability, and ease of use to reduce dependency on specialist operators.^56^

#### 3.2.1 Portability and power autonomy

True portability in LREs means more than just a small size and low weight, it requires functional autonomy from unreliable infrastructure.^57^ The model POC device must be a self-sufficient analytical system, a requirement that requires strict power budgets.

**Ultra-low power architecture.** Engineering begins at the element level, selecting microcontrollers and sensors that operate in ultra-low-power sleep modes. System design must be optimized to reduce the use of high-drain components, such as pumps or heaters, using pulsed operation and predictive control. Passive microfluidics, which utilize capillary forces rather than active pumps, exemplify power-aware design.^58^^–^^61^
**Adaptive power sourcing.** The device’s power system must be agnostic to its energy input. This demands robust circuitry capable of accepting input from a wide range of sources, including unstable grid power, vehicle batteries, solar panels, and hand-crank generators.^62^ This flexibility guarantees functionality across the diverse infrastructural environments of LREs, from clinics with irregular electricity to remote villages that are completely off-grid.

#### 3.2.2 Holistic robustness and environmental hardening

Devices designed for LREs must be engineered to endure a "trials-by-use" lifecycle far more demanding than that of a clinical lab. Thus, devices must be engineered for multi-axial robustness, incorporating thermal, mechanical, and operational durability. Mechanical and environmental resilience, engineering for robustness means foreseeing a lifetime of mechanical shock from transit over unpaved roads, exposure to pervasive dust and moisture, and substantial temperature variabilities.^63^ This requires the use of Design for Assembly (DfA) and Design for Manufacturing (DfM) principles, which favour monolithic, over-molded casings with high ingress protection (IP67+), reduced part counts, and polymers chosen for high impact strength, UV tolerance, and compatibility with chemical disinfectants.^64^

**Operational stability and error mitigation.** True robustness extends to the avoidance of user error.^65^ This is achieved through poka-yoke (mistake-proofing) principles, which involve designing consumables and sample interfaces that fit only one way, utilizing engraved sensors to detect sample presence before initiating a test, and constructing fluidic paths resistant to bubbles or clots.^66^ The device is designed to reflect a clear error message whenever a misstep occurs, not a corrupted test or broken device.

#### 3.2.3 Cognitive simplicity and user-centric design

It is important to recognize that the end-user in LREs is often a community health worker with minimal technical training, operating under time constraints and challenging conditions. Thus, the interface, both digital and physical, must be designed for foolproof operation with minimal cognitive load.^67^ The ideal assay protocol should need fewer than three core steps, encompassing a ’sample in, answer out’ philosophy. This forces the integration of complex procedures such as reagent handling, timing, and washing steps into a single, self-sufficient cassette or cartridge.^68^ This compels the integration of complex procedures, including reagent handling, timing, and washing steps, into a single, self-sufficient cartridge, or cassette. The device’s physical form factor must give clear tactile feedback and be easily manipulated by users with diverse physical abilities.^69^

**Ambient intelligence and autonomous operation.** The device’s internal intelligence must compensate for the lack of a specialized operator. This can be accomplished through automated calibration, internal quality controls that run with every test, and environmental sensors to flag out-of-spec conditions.^70^ The goal is to shift the analytical burden from the operator to the instrument, thereby ensuring objective and reliable results regardless of the user’s skill level.

### 3.3 Power and Optical Component Optimization under Field Conditions

The evolution of a POC diagnostic from a coordinated laboratory environment to the variable and often severe field conditions represents a crucial point where theoretical performance meets practical reality.^71^ The consistency of the device is eventually influenced by the efficiency and resilience of its main subsystems, with photonic elements and power construction being vital. Optimization in this situation is not simply an improvement but a crucial necessity for practical viability, requiring a co-design strategy that combines performance specifications with infrastructural and environmental constraints.^72^^,^^73^

#### 3.3.1 Photonic system engineering for environmental stability

The optical detection method including absorbance, scattering, chemiluminescence and fluorescence, are pillars of POC diagnostics owing to their high potential for quantification and sensitivity.^71^ Conversely, their performance is highly prone to the field environment, requiring robust engineering approaches.

**Spectral efficiency and signal-to-noise ratio improvement.** The primary goal is to maximize the signal-to-noise ratio (SNR) while minimizing power consumption. This starts with the intentional choice of components.
**Light sources.** Light-emitting diodes (LEDs) are preferred for their minimal power consumption, extended lifetime, rapid switching capabilities, and monochromaticity.^74^ Their intrinsic stability reduces the need for complex referencing techniques. Laser diodes may be utilized for applications that require high spatial consistency, but at the cost of increased power consumption and enhanced thermal management requirements.
**Detectors.** Silicon photodiodes are characterized by a highly linear response, robustness, and low cost. For lower light levels, miniature photomultiplier tubes (PMTs) or avalanche photodiodes (APDs) may be used; however, they require more complex biasing circuits.^75^^,^^76^ Integration of low-noise amplification and filtering electronics with the detector is essential to minimize signal degradation.
**Optical path design.** A minimalist optical train is crucial. Approaches involve utilizing high-quality optical filters with sharp cut-on or cut-off edges to block background light and using locked-in amplification matched with a modulated LED source to differentiate the signal from ambient optical noise. The entire optical path should be sealed to prevent contamination from moisture and dust, which can potentially scatter light and introduce substantial measurement errors.
**Resilience to ambient perturbations.** An essential requirement for any diagnostic device intended for global health is its ability to perform consistently despite vast differences in its operating environment.^73^ It must deliver the same level of accuracy and reliability whether it sits on a bench in a humid, sun-drenched tropical clinic or in a weakly lit room at a remote field station. Achieving this begins with robust environmental shielding, a purpose-designed physical enclosure that completely seals the optical detection chamber from all ambient light is a crucial, non-negotiable foundation. Beyond this, the device must also contend with the more insidious effects of temperature. The output of critical components, such as LEDs and photodetectors, is inherently temperature-dependent, leading to potential drift in readings. This can be effectively managed by integrating low-power temperature sensors and applying software-based correction algorithms or by utilizing constant-current drivers with optical feedback, thereby ensuring stable performance across a wide range of temperatures. Finally, long-term resilience requires proactive strategies against component degradation. Optical surfaces are susceptible to fungal growth, fogging, and dust accumulation, which can gradually obscure signals. Employing a combination of hydrophobic coatings, hermetic seals, and easily cleanable or even consumable optical windows will safeguard the device’s integrity, promoting extended and reliable service life in the most challenging environments.

#### 3.3.2 Power system architecture for infrastructure-independent operation

The unreliable electricity supply from the grid in low-resource settings (LREs) lifts power management from a secondary concern to a primary design consideration. The system should be designed for maximum energy efficiency and flexibility in sourcing.

**Hierarchical power management.** A complex power management-integrated circuit (PMIC) is the foundation of an effective design.^62^^,^^77^^,^^78^ It must implement a hierarchical strategy:
  • Ultra-low-power sleep mode. The device must spend the great part of its time in a state using nominal current (e.g., <100  μA), running up following operator interaction or a pre-set timer.
  • Dynamic power scaling. The processor, peripheral voltages, and clock frequencies must scale dynamically with the computational load.
  • Pulsed activation of high-load components. Power-intensive elements such as cartridge heaters for isothermal intensification (e.g., LAMP, RPA) or motors must be stimulated in short, high-current pulses instead of continuously, with their power draw carefully sequenced to circumvent overloading the system.
**Adaptive and redundant energy sourcing.** The device should be agnostic to its energy source to guarantee functionality across a wide range of field settings.
**Multisource input.** The power input stage must be designed to take a diverse range of voltages (e.g., 5V-18V) from sources such as 12V car batteries, AC adapters, and portable solar panels.^62^
**Smart battery buffering.** An internal, high-energy-density lithium-polymer or lithium-ion battery serves as a crucial buffer. The system must be capable of functioning merely from this battery while concurrently managing its charge cycle from any accessible external source. Advanced fuel-gauging algorithms can provide operators with precise estimates of remaining fuel or operational time, a critical element for planning in off-grid environments.

### 3.4 Data Acquisition and Mobile-Based Readouts

The game-changing capability of POC diagnostics is fully realized only when a test result is correctly interpreted and integrated into the clinical or public health decision-making process. The union of POC devices with mobile technology and digital health platforms signifies a framework shift, transforming inaccessible diagnostic events into interconnected data nodes within a wider health intelligence network.^76^^,^^79^^–^^81^ This incorporation, which encompasses data acquisition, objective readout, and secure transmission, is crucial for enhancing diagnostic accuracy, enabling real-time shadowing, and strengthening health systems in LREs.

#### 3.4.1 Mobile platforms as universal analytical instruments

The pervasive global penetration of mobile phones, including smartphones, provides an unprecedented opportunity to leverage their sophisticated hardware as a versatile readout platform for POC diagnostics.^82^^–^^84^

**Camera-based quantification.** The smartphone camera can be used as a high-resolution spectrophotometer or imager.^85^ For colorimetric assays such as lateral flow assays and microfluidic paper-based analytical devices (μPADs), it can quantify colour intensity. For fluorescence-based assays, it can detect emission intensity, whereas for chemiluminescence, it can measure light output. Advanced image processing algorithms, which correct for ambient light variations, nonuniform illumination, and perspective distortion, are utilized to transform a raw image into a quantitative result.
**Alternative sensor interfacing.** Outside the camera, other built-in sensors can be reused for additional functionality. The USB-C port or audio jack can offer power and a data channel for simple peripheral sensors (e.g., for electrochemical detection). The phone’s accelerometer can be utilized in assays based on magnetic particle clumping, measuring the change in oscillation frequency.^83^ Enhancing diagnostic accuracy and accessibility. Mobile readouts alleviate the prejudice of visual interpretation, reducing inter-user variability and defining exact cut-off values between positive and negative results. This is predominantly critical for semiquantitative tests (e.g., determining weak positive lines) and multiplexed assays. Moreover, by embedding the test analysis logic within a dedicated application, the system can guide the operator through the entire testing process with audiovisual prompts, making complex tests accessible to lay health workers.

#### 3.4.2 Data acquisition, connectivity, and the creation of health intelligence

The full power of mobile-integrated diagnostics lies in their ability to transform a local result into a global data point, enabling a transition from passive testing to active health management.^86^^,^^87^

**Structured data capture and connectivity.** The evolution of modern POC diagnostics extends far beyond generating a simple positive or negative result. Today’s connected POC devices and their companion mobile applications are designed to capture a rich, structured dataset that provides critical context and ensures traceability. This metadata characteristically comprises several key elements. First, comprehensive test information is logged, including the reagent lot number and expiration date, to ensure quality. In addition, the device’s internal quality control status is verified, which confirms the assay’s validity. Second, essential patient and operator data are captured, which may include a unique patient identifier for the medical record, an operator ID to assign responsibility, and geographic location data obtained via GPS or network triangulation for epidemiological mapping. Finally, the system records temporal and contextual data, such as a precise timestamp of the test and, if the device is equipped with environmental sensors, ambient conditions such as temperature and humidity that could impact the result. This holistic data capture transforms a simple test result into a powerful, auditable data point for clinical decision-making and public health surveillance. This structured data can be transmitted in near real-time through cellular networks (4G/GSM/3G), Bluetooth, or WIFI to centralized cloud-based health information systems, such as laboratory information management systems (LIMS) or district-level health dashboards.
**Enabling syndromic surveillance and system strengthening.** The combination of decentralized POC data makes a powerful surveillance tool.
**Instantaneous outbreak detection.** The geospatial and temporal mapping of disease-specific positives (e.g., for influenza or malaria) can show emerging outbreaks days or weeks before conventional reporting mechanisms, enabling a quick public health response.
**Program management and resource optimization.** Health authorities can monitor testing rates, positivity rates, and stock levels of tests and consumables across various facilities. This data-driven insight permits vigorous resource allocation, predictive supply chain management, and targeted training and supervision. Clinical decision support and patient navigation-positive results can automatically prompt follow-up actions within the digital system, such as notifying a supervising clinician, generating a referral for confirmatory testing or treatment, or enrolling a patient in a remote adherence monitoring program for chronic diseases such as HIV.

Although the engineering, optical, power management, and digital connectivity techniques discussed above are largely applicable to POC diagnostics intended for low-resource environments, their successful implementation hugely relies on the healthcare systems in which they function.

### 3.5 Implementing Biophotonics-Based Point-of-Care Diagnostics Within the South African Healthcare System

Although the preceding sections have discussed the engineering principles, power management strategies, optical system optimization, and digital connectivity needed for POC diagnostics in resource constrained environments, successful implementation largely depends on the healthcare system in which these technologies are deployed. South Africa represents particularly essential case study because it has one of the largest and most advanced healthcare systems in sub-Saharan Africa yet continues to face huge healthcare inequalities. The country operates a dual healthcare system constituting a well-resourced private sector serving a minority of the population alongside an overburdened public sector that offers care for the majority. Moreover, South Africa faces a well-recognised quadruple burden of disease comprising of HIV/AIDS and tuberculosis, rising noncommunicable diseases, maternal and child health challenges, injuries, and violence.^88^ These healthcare challenges are further worsened by lack of skilled healthcare professionals, uneven distribution of laboratory infrastructure, delayed access to centralized diagnostic services in rural settings, equipment maintenance difficulties, intermittent electricity supply, and logistical barriers affecting specimen transport and reporting.^5^^,^^88^

Although the National Health Laboratory Service has substantially strengthened diagnostic capacity, many primary healthcare clinics continue to benefit from decentralized testing that offers rapid clinical decision-making closer to the patient. Inherently, the successful deployment of biophotonics-based POC diagnostics in South Africa needs more than analytical performance alone. Devices must be adapted to local clinical workflows, national treatment guidelines, workforce capacity, maintenance systems, procurement pathways, and digital health infrastructure to ensure long-term sustainability.^89^^–^^94^

Against this background, the subsequent subsections discuss three key implementation pillars within the South African healthcare system, deep localization of diagnostic technologies, healthcare workforce capacity building, and sustainable maintenance and technical support ecosystems.^88^ Addressing it needs a continual, three-pronged strategy: (1) deep localization, (2) efficient capacity building, and (3) a strong maintenance ecosystem.

#### 3.5.1 Deep localization: beyond linguistic translation to clinical and operational integration

Localization is a multifaceted process that expands far beyond the translation of operator interfaces into local languages. It is the complete adaptation of the technology and its related workflows to the exact clinical, regulatory, and cultural context.

**Clinical pathway integration.** A diagnostic result is only as valuable as the clinical action it prompts. A key aspect of localization is ensuring that POC test output are contextualised within South Africa’s standard treatment guidelines and essential medicines list (EML).^89^ For instance, a CD4 count or viral load result from a POC device must precisely inform the specific antiretroviral therapy (ART) initiation or switching protocols recommended by the National Department of Health. This warrants that the technology directly enables clinicians at the primary care level to make evidence-based decisions without uncertainty.
**Workflow and ergonomic adaptation.** The design of the device must be guided by the physical realities of a South African primary healthcare clinic, which may be congested, short-staffed, and constrained by bench space. Noise level, form factor, and assay time must be optimized to prevent disrupting clinic flow. In addition, the practical approach to the Care Kit (PACK) program, established in South-Africa and successfully expanded to countries such as Brazil, demonstrates a mentorship framework for integrating new tools into current clinical practice, representing how to incorporate new diagnostic algorithms into established platforms for managing common conditions.^90^^,^^91^

#### 3.5.2 Capacity building: from task-shifting to skill-enabling

The implementation of a new technology must be accompanied by a long-term commitment to staff training. This moves beyond basic "task-shifting" to a more complex model of "skill-enabling," building confidence and competence. A standardized, off-the-shelf training approach is fundamentally ineffective for the diverse roles within a healthcare system. Success, instead, depends on a stratified model that delivers tailored instruction to different levels of the healthcare workforce. For frontline operator users such as nurses and community health workers, training must be intensely practical and hands-on. Thus, building the technical capability for the entire testing process, from correct sample collection and device operation to accurately interpreting results and performing basic troubleshooting. Utilizing train-the-trainer models and peer-to-peer learning, as exemplified in initiatives like the PACK rollout, is key to enhancing both the scalability and long-term sustainability of this skills development.^44^ For supervisors and facility managers, the focus of training must shift from technical operation to holistic oversight. Their training must focus on interpreting consolidated data for public health action, managing quality assurance procedures, overseeing supply chain logistics, and coordinating patient referrals based on the POC results produced by their teams. Disapprovingly, initial training is inadequate on its own. Continuous learning and quality assurance are critical to uphold high standards. This necessitates a continuing commitment to professional development, reinforced by digital job aids, remote mentoring through telehealth platforms, and regular skill testing. This continuous support system is essential to prevent the inevitable drift in procedure and knowledge that occurs over time, ensuring reliable and consistent patient outcomes.

#### 3.5.3 Engineering a sustainable maintenance environment

The silent graveyards of abandoned medical equipment across sub-Saharan Africa are stark evidence of the fatal flaw of neglecting maintenance.^92^^,^^93^ To break this cycle, a proactive, system-oriented strategy is essential, built on three core pillars. First, manufacturers must embrace design for serviceability (DfS), prioritizing repairability from the outset.^54^^,^^94^ This involves creating devices with a modular design, comprising simply swappable components such as heating elements or optical blocks that can be substituted at the district level without requiring the entire device to be returned. Moreover, embedded self-diagnostic capabilities can indicate failures to specific subassemblies, while accessible, simplified documentation empowers technicians with clear repair manuals and fault-finding charts. Second, building a National Technical Workforce is a non-negotiable long-term solution. Relying solely on international service contracts is unsustainable. A more resilient model involves partnering with South Africa’s network of Technical and Vocational Education and Training (TVET) colleges to design accredited biomedical technician programs. This creates a local team of professionals skilled in repairs, calibration, and preventive maintenance, fostering local ownership and reducing dependency on external vendors. Finally, a functioning device is useless without a reliable supply chain. Strengthening the logistics for spare parts and consumables is important. This requires formally integrating POC test cartridges and reagents into national tender procedures and leveraging established, successful logistics networks such as those for HIV/AIDS or TB medications to guarantee a consistent "last-mile" delivery to even the most remote clinics. Only by integrating durable design, local technical capacity, and robust supply chains can we replace the equipment graveyards with sustainable, life-saving healthcare systems.

## 4 Advances in Biophotonics Technologies for Point-of-Care Testing

POC testing (POCT) is medical diagnostic testing performed at proximity where a patient is receiving care resulting to a possible change in the care of the patient.^95^^,^^96^ These tests may be performed outside the main clinical laboratory, at the "point of care” such as doctor’s consultation room, hospital bedside or in a mobile clinic. An approach enabling faster results, leading to robust diagnoses, eliminating the need to transport samples and waiting for lab turnaround times.^97^ Rapid results allow healthcare providers to initiate immediate treatment suggestions during the patient’s encounter, leading to a more efficient and targeted care process in real-time. POC testing includes a wide variety of test, similar to simple home tests such as pregnancy tests, blood glucose monitoring tests, HIV rapid tests, to more complex tests performed by healthcare professionals in clinical facilities using portable devices, such as blood gas analysis or rapid COVID-19 antigen tests.^98^ Advantage of such testing occurs in a variety of settings outside of a central laboratory and is especially valued in regions with limited access to conventional laboratories, improving diagnosis and treatment for diseases.

Recent advances in miniaturized optics, photonic materials, and microfabrication have transformed photonics diagnostics from laboratory instruments into portable, field-ready POCT systems. These innovative systems have integrated sensing, signal processing, and connectivity into portable devices, enabling rapid accurate and user-friendly detection of diseases directly at POC.^99^ A crucial goal for improving health equity and resilience in resource-limited settings. The convergence of miniaturized optics, smartphone computation, and low-cost materials is making photonics POC diagnostics increasingly viable in the real-world in low-resource contexts.^100^ These technologies are not only improving access to high-quality diagnostics but also creating new opportunities for local innovation and manufacturing in African health systems. Since 1962 to date, these innovations have been increasingly changing the world of POCT, with portable devices such as smartphone biosensors, spectrometers, and colorimetric analysers. Table 1 gives a summary of some of the technologies with their best use cases for POCT deployment.

**Table 1:** Summary of biophotonics technologies for POC testing.

Technology	Advantages	Applications
Smartphone biosensors	Low cost	Rapid tests for biomarkers and pathogen detection.
	Battery powered device	
	High accessibility	
	Integrated data transmission for telemedicine	
	Operable by minimally trained health workers	
	Moderate to high sensitivity	
	Excellent portability	
Portable Raman/fluorescence spectrometers	Moderate cost	Pathogen identification, drug detection, hemoglo-bin assays
	Battery-powered operation allowing moderate power usage	
	Rugged casings	
	Instant wireless reporting make these instruments ideal for remote screening and environmental surveillance.	
	Very highly sensitive	
	High portability	
LED-based modules	Low cost	Colorimetry, reflectance, imaging
	Low power consumption	
	Compatibility with disposable cartridges	
	Safe for skin-contact and *in vivo* applications	
Paper/polymer sensors	Ultra-low cost	Field assays, disposable tests
	Easy disposal (biodegradable)	
	No external pumps or power requirements	
	Ideal for one-step assays and environmental or infectious disease screening	
	Moderate sensitivity	
	Excellent portability	
Fiber/plasmonic/nanophotonic sensors	Moderate cost	Label-free, multiplex assays, biomarker detection, environmental pathogen monitoring, and *in vivo* biochemical sensing
	Low to moderate power requirement	
	Small footprint	
	Potential for multiplex detection	
	Compatibility with LED or laser diode illumination	
	Very high sensitivity	
	High portability	
Wearable/implantable optics	Moderate cost	Chronic monitoring, telehealth
	Low power requirement	
	Real-time, longitudinal data collection	
	Noninvasive or minimally invasive operation	
	Wireless communication for telemonitoring	
	Moderate sensitivity	
	Continuous excellent body fit	

### 4.1 Smartphone-Integrated Optical Biosensors

Due to high-resolution cameras, light sources, displays, processing power, and the capabilities for wireless communication have advanced smartphones to powerful analytical platforms for rapid pathogen detection (e.g., malaria parasites and HIV viral load proxy tests), environmental toxin monitoring, and mobile cytometry for field diagnostics.^101^ The integration of smartphones with optical modules such as lenses, LEDs, filters, and microfluidic cartridges has allowed them to function as spectrometers, microscopes, and colorimetric analyzers fitted with fluorescence, colorimetric, SPR/fibre, and Raman readouts with 3D-printed cradles or clip-on optics.^102^ Examples include spectroscopy-on-phone, which utilizes compact diffraction gratings or Fabry-Pérot filters coupled to the camera, enabling reflectance, fluorescence, or absorption measurements.^103^ Fluorescence readers with clip-on modules with excitation LEDs and emission filters capable of detecting biomarker fluorescence or quantum dot signals.^104^ SERS combines molecular fingerprint specificity with potential single-molecule sensitivity.^105^ SERS-on-chip with nanostructured plasmonic substrates can convert the smartphone camera into a portable Raman analyser.^106^ Smartphones with AI-assisted applications, featuring mobile AI algorithms that can interpret optical patterns, enable automated diagnostics for malaria, TB, HIV, and COVID-19 antigen detection.^79^^,^^107^^–^^110^

### 4.2 Portable Raman, Fluorescence, and Spectroscopic Systems

The advancement of spectrometers to handheld and portable benchtop systems fitted with miniaturization of lasers, filters, and complementary metal-oxide-semiconductor (CMOS) spectrometers, such as Raman and fluorescence systems, with analytical performance such as that of laboratory units, has made things easier to work at POCT.^111^ In addition, portable SERS platforms can detect pathogens, metabolites, or counterfeit drugs at nanomolar levels.^112^^,^^113^ Fiber-coupled Raman probes permit non-invasive tissue analysis, such as cervical^114^ or oral lesions,^115^ at POCT.

Fluorescence spectroscopy, utilizing compact diode-based excitation and smartphone readout modules, supports immunoassays and nucleic acid detection.^116^ Time-resolved fluorescence improves background rejection in field testing. Absorption and reflectance spectroscopy with LED/photodiode pairs enable simple colorimetric or haemoglobin assays.^117^ Multispectral reflectance systems quantify jaundice, anemia, or tissue perfusion.^118^^–^^121^ Battery-powered operation, rugged casings, and instant wireless reporting make these instruments ideal for remote screening and environmental surveillance, which are POCT benefits.^97^^,^^122^ State-of-the-art SERS-LFIA hybrids coupled to bespoke handheld readers show strong promise for POC protein/toxin detection.^123^^,^^124^

### 4.3 LED-Based Optical Detection and Imaging Modules

LEDs are compact, efficient, and inexpensive light sources that have been spectrally tailored for diagnostic use in the integration into POC systems enabling both excitation and illumination functions^125^ These LEDs (UV–NIR) are now standard exciters for compact fluorescence/absorbance readers and smartphone add-ons due to low power cost and multiwavelength arrays.^74^ Examples include multiwavelength LED arrays which allow sequential or multiplexed excitation for fluorescence and absorbance assays.^126^^,^^127^ Pulsed LED sources support time-gated detection and fluorescence lifetime imaging (FLIM), and LED-based reflectance imagers assess skin and mucosal tissue conditions, such as pallor, cyanosis, and wound healing.^128^^,^^129^ The advantages of these modules are low power consumption, compatibility with disposable cartridges, and safety for skin contact and *in vivo* applications (Table 1). These modules have been utilized in low-resource settings, including LED colorimetric readers for lateral flow assays, bilirubin meters for neonatal care, and low-cost imaging tools for dermatological screening.^118^^,^^130^ The greatest advantage of fluorescence readers using LEDs is that they routinely achieve a limit of detection range in the nM to pM, depending on the assay chemistry, whereas absorbance modules tend to be sensitive to μM to nM levels.

### 4.4 Paper- and Polymer-Based Photonic Biosensors

Paper and polymer substrates offer an affordable platform for integrating optical sensing elements and fluidics into disposable, lightweight devices.^131^^,^^132^ They are particularly suitable for resource-limited environments where single-use and instrument-free assays are preferred. Technological advances include μPADs (microfluidic paper-based analytical devices), which combine capillary-driven flow with colorimetric or fluorescent detection.^133^ Polymer-based waveguides and photonic crystals with the capacity to improve sensitivity and allow coupling Venugopalan with external light sources.^134^ Paper plasmonic composites that embed nanoparticles for enhanced Raman or plasmonic colorimetry.^135^ Hybrid smartphone readouts that use cameras to quantify optical intensity changes or spectral shifts.^98^^,^^136^ The advantages of POC include ultra-low cost and easy disposal (biodegradable), as well as no external pumps or power requirements. This makes them ideal for one-step assays and environmental or infectious disease screening (Table 1). Other examples include the paper-based ELISA for HIV and hepatitis, polymer photonic sensors for glucose or uric acid, and hybrid paper–microfluidic colorimetric tests for malaria antigens.^137^

### 4.5 Fiber-Optic, Plasmonic, and Nanophotonic Detection Strategies

Miniaturized optical fibres and nanostructured photonic elements are enabling unprecedented sensitivity and multiplexing in compact formats.^138^ The likes of fiber-optic sensors that leverage evanescent-field sensing, Bragg gratings, or interferometry for real-time biochemical monitoring and can be embedded in catheters, wound dressings, or microfluidic chips for POCT.^139^^–^^141^ Plasmonic sensors that use metal nanostructures (Au, Ag) to amplify local electromagnetic fields for SPR/LSPR-based sensing and are highly sensitive to changes in refractive index, which are best for detecting low-concentration biomarkers.^142^ Portable plastic optical fibres with SPR coatings and smartphone-coupled readouts are being piloted for POC inflammatory/cancer biomarkers.^143^ Nanophotonic resonators featuring silicon, gallium nitride (GaN), or polymer micro rings and meta-surfaces offer ultra-compact, label-free biosensing with scalable integration capabilities, making them suitable for mass production through CMOS fabrication.^144^^–^^146^ Their advantages for POCT include high sensitivity and a small footprint, potential for multiplex detection, and compatibility with LED or laser diode illumination. Applications have included early disease biomarker detection, environmental pathogen monitoring, and *in vivo* biochemical sensing.

### 4.6 Emerging Wearable and Implantable Optical Sensors

Advances in flexible photonics, biocompatible materials, and wireless power transfer have accelerated the field of POCT systems, enabling the progression of wearable and implantable optical sensors for continuous health monitoring.^98^ These biosensors naturally noninvasive and provide real-time monitoring for the patient at any given point-in-time.^147^ Commercially available wearable sensors include wristwatches, eyewear, facemasks, skin patches, clothing, and footwear that utilize several biofluids, consisting of numerous sensing platforms based on scattering, reflection, absorption, and interference, including sensor modalities based on various phenomena.^148^ Eyewear sensors include smart contact lenses used for continuous eye health monitoring.^149^ Skin patches include flexible, skin-mounted optical sensors for monitoring heart rate and oxygen saturation (${\mathrm{SpO}}_{2}$),^150^ glucose,^151^^,^^152^ and hydration.^153^ Photoplethysmography (PPG) and reflectance spectroscopy are integrated into wristbands and patches for detecting physiological changes in response to human skin types.^154^ Stretchable optoelectronic films for wound oxygenation monitoring.^155^ Clothing and footwear biosensors.^156^^–^^159^ Although implantable sensors include biodegradable optical fibres and hydrogel waveguides for *in vivo* glucose, lactate, or pH detection.^140^^,^^159^ In addition, optical resonators and plasmonic implants for continuous biochemical tracking in deep tissues.^160^ These are noninvasive or minimally invasive operations provide real-time, longitudinal data collection, and wireless communication for telemonitoring.^161^

Wearables and implantable optics are transforming the management of chronic diseases (hypertension, diabetes, HIV) through low-cost, mobile-linked monitoring, supporting preventive healthcare and reducing hospital burden.^140^

## 5 Applications in South African and Sub-Saharan Health Contexts

### 5.1 Infectious Disease Diagnostics

Biophotonics technologies in POC diagnosis have the potential to detect and manage infectious diseases in South Africa and the sub-Saharan countries. This technology has the potential to address significant health issues related to diseases that are prevalent in low-resource settings which will be discussed in this section.

#### 5.1.1 Tuberculosis and HIV co-infection

The high prevalence rates of TB and HIV co-infection poses a great challenge and compromises the health landscape in South Africa. Biophotonics methods have emerged as robust methods in POC diagnostics due to the challenges posed by TB and HIV co-infections in these low-resource settings. Treatment protocols are complicated due to the interplay and high burden of these two infections. In South Africa, the burden of multidrug-resistant TB (MDR-TB) is acute among the population. Studies have shown that untreated drug-resistant strains of TB cause high death rates in immunocompromised TB patients with HIV.^162^^,^^163^ The introduction of POC diagnostics, which can smoothly provide results, is urgently needed to improve patient management in such settings. Conventional diagnostic methods are slow and lack the accessibility required for timely intervention.^164^^,^^165^ Urine-based tests such as the lipoarabinomannan assay have recently been used for immunocompromised patients as POC.^166^^,^^167^ These tests are rapid and sensitive for immunocompromised patients who have difficulty with sputum-based testing. A previous study demonstrated that this test has the potential to enhance TB detection in severely immunocompromised patients, where traditional methods are ineffective.^168^ This observation calls for the urgent need to develop and deploy a robust POC method to improve case detection and treatment outcomes. There is a need to integrate biophotonics into the diagnostic space to streamline workflows at healthcare centres, thereby reducing delays compared to traditional methods.^168^^,^^169^ Photonics POC tools are rapid and require minimal infrastructure, thereby increasing access to care in low-resource settings.^170^^,^^171^ The move to biophotonics tools is relevant to South Africa, where resource constraints and logistical challenges significantly affect patient care.

#### 5.1.2 Malaria and other vector-borne diseases

The use of biophotonics-based POC is an emerging tool in the context of malaria and other vector-borne diseases. Malaria is endemic in sub-Saharan Africa and is difficult to diagnose in asymptomatic cases and presents a major public health challenge. Conventional Rapid Diagnostic Tests (RTDs) are ideal in high-transmission areas and have been extensively used in detecting malaria caused by *Plasmodium falciparum*.^172^ These tests are not effective in low-transmission settings, as they miss some infections, as shown by a study conducted in KwaZulu-Natal, South Africa.^172^ A robust method is therefore needed to complement the existing RTDs in detecting both symptomatic and asymptomatic malaria. Diagnostic sensitivity is improved using biophotonics-based POC diagnostic techniques.^173^ Biophotonics can detect low levels of parasites, thereby disrupting the transmission of malaria in these endemic regions. Fast and reliable results can be provided by POC with smartphone-integrated devices in low-resource settings, bringing timely treatment.

#### 5.1.3 COVID-19 and respiratory infections

The rapid implementation of POC diagnostic tests has been valuable in the management of public health response during the COVID-19 pandemic. The POC tools have resulted in rapid testing and surveillance in South Africa and other sub-Saharan African countries. A major promising area of photonics has been the use of low-cost nanoplasmonic sensors for detecting SARS-CoV-2. The development of luminescent biosensors for POC SARS-CoV-2 detection show much promise in enhancing diagnostic capabilities in diagnostic technologies.^174^ These innovations facilitate rapid testing in community health settings, allowing for efficient management of the pandemic. The use of chest computed tomography (CT) imaging has been explored as a supplemental diagnostic tool to identify pneumonia associated with COVID-19, particularly when RT-PCR results are negative.^175^ Accessible, affordable, and reliable diagnostic methods are required to ensure timely and accurate testing in low-resource settings.^5^^,^^176^ Biophotonics-based POC diagnostics have the greatest potential to manage infectious diseases in South Africa and sub-Saharan Africa. These technologies can be easily integrated into existing healthcare settings to improve health outcomes for individuals.^170^

### 5.2 Noncommunicable Diseases

#### 5.2.1 Cancer screening and optical biopsy

The high incidence of cancer reflects an urgent need for a robust cancer screening and diagnostic tool in South Africa and other sub-Saharan countries. Conventional cancer screening methods are often ineffective and rely heavily on the availability of trained personnel and adequate laboratory infrastructure. These facilities are limited in low-resource settings.^177^ Biophotonics are used as POC diagnostics in cancer screening and optical biopsy in South Africa and sub-Saharan Africa. These regions have a high burden of noncommunicable diseases, hence the pressing need for the development of an accessible and affordable diagnostic tool. A promising emerging application of biophotonics in cancer diagnostics is the use of portable optical imaging system. Previous studies have demonstrated that optical coherence tomography and optical biopsy are feasible for deployment in resource-limited settings, thereby improving access to cancer screening.^178^ The combination of direct cytology and AI provides a promising method for screening cancer. A study in Kenya addressed the shortage of pathologists due to this method.^179^

#### 5.2.2 Diabetes and cardiovascular disease monitoring

Diabetes and cardiovascular diseases (CVD) face challenges in South Africa and the sub-Saharan Africa due to limited access to healthcare and poor infrastructure. However, the development of biophotonics POC diagnostics is a promising avenue. The prevalence of diabetes in South Africa is high, with many people remaining undiagnosed. Biophotonics-based POC diagnostics can overcome these challenges. Optical glucose sensing can enable early identification and routine monitoring.^180^ Photonics innovations give timely feedback to patients and healthcare professionals. Hypertension is a major risk factor causing morbidity and mortality in South Africa.^181^ The prevalence of hypertension in South Africa is expected to rise, presenting the need for improved monitoring and management strategies.^182^ The integration of biophotonics can result in real-time blood pressure monitoring through portable devices. This integration offers practical solutions in rural and urban areas.^182^^,^^183^

### 5.3 Maternal, Neonatal and Rural Health Applications

The use of biophotonics-based POC diagnostic tools in maternal and neonatal health in low-resource settings is projected to improve as there is a limited access. Biophotonics are widely used in the development of cheap diagnostic tools that can monitor conditions associated with pregnancy. Traditional birth attendants play a pivotal role in maternal care as they know how to recognize certain symptoms related to maternal health, especially in rural areas. This is, however, limited by the lack of necessary equipment, which leads to severe outcomes as referrals do not occur on time.^184^ Biophotonics-based POC diagnostics help traditional birth attendants in their ability to screen for at-risk pregnancies, thereby improving maternal outcomes.^185^ Handheld ultrasound imaging techniques are used in the early detection of fetal growth restrictions and ectopic pregnancies. These devices can streamline antenatal care, thereby allowing healthcare workers to conduct assessments in remote areas where access to specialists is limited.^185^^,^^186^

### 5.4 Environmental and Waterborne Pathogen Detection

There is a growing concern about the quality of water in the resource-limited settings in South Africa and other parts of sub-Saharan Africa. This concern highlights an urgent need to develop innovative solutions for detecting these pathogens, as they cause waterborne infections. There must be robust diagnostic tests that can be deployed in the field. The use of traditional methods to detect pathogens in water is less effective. These methods are not ideal in a rural setting as they require more time and laboratory infrastructure.^187^^,^^188^ Photonics-based diagnostic tools such as optical biosensors can offer quick and reliable detection of these pathogens. The incorporation of nanotechnology in biosensors offers an enhanced detection of these pathogens. The integration of nanomaterials and traditional methods increases the detection limit for pathogens in low-resource settings.^187^ The application of biophotonics-based diagnostic tools aids in monitoring environmental health and detecting diseases.

### 5.5 Veterinary and Agricultural Applications Relevant to One Health

The One Health approach is being recognized in the application of biophotonics in the veterinary and agricultural space. The application of biophotonics POC diagnostics provides solutions for detecting pathogens and managing diseases in livestock and wildlife. Furthermore, these technologies improve agricultural productivity and food safety in South Africa and sub-Saharan Africa. Biophotonics-based techniques are overtaking the space in the rapid detection of zoonotic pathogens, where CRISPR technology is entirely utilised.^189^ Biophotonics POC diagnostic tools can be used to enhance the surveillance and monitoring of diseases in livestock. Furthermore, these technologies can be used in veterinary settings to detect *Schistosoma hematobium* using CRISPR technology.^190^ The use of these tests is important as they inform control measures, which result in improved health for livestock, thereby minimizing economic losses in farming communities. The Loop-mediated isothermal amplification method combined with CRISPR is used to identify malaria. This technique is adapted to detect various disease vectors that could cause emerging zoonotic diseases.^191^ Biophotonics technologies can be applied to monitor infection rates and facilitate veterinary healthcare. Another critical area in the agricultural space is the monitoring of antibiotic resistance in pathogens from environmental sources and livestock. Surveillance is of utmost importance because antibiotic resistance is a common phenomenon. Antibiotic resistance has a direct impact on food safety and public health.^192^ The implementation of biophotonics-based diagnostics improves surveillance efforts and promotes the use of antibiotics by farmers.

## 6 Data Analytics and Digital Health Integration

Digital health innovations continue to reshape the global healthcare system by enhancing access, efficiency, and quality of care.^193^ Technologies such as AI, mobile health applications, big data analytics, and cloud-based telemedicine have been widely used to improve clinical decision-making. The convergence of these technologies has led to robust, decentralized diagnostic, and data management ecosystems.^193^ Particularly important for developing countries, where infrastructure variability, cost constraints, and regulatory oversight are persistent challenges. This section presents current research and practices in digital health innovations, mainly highlighting the deployment of AI, edge computing, and cloud-based platforms, challenges encountered, and directions for future research.

### 6.1 AI-Assisted Interpretation of Optical Signals

Recent advancements in optical sensing (fluorescence detection, colorimetric lateral-flow assays, and plasmonic nanosensors) have been integrated with AI to improve accuracy and reliability. This is due to the fact that machine learning (ML) and deep learning (DL) algorithms have the potential to convert raw optical signals into qualitative readout for POC applications.^194^ ML is a subset of AI that uses algorithms to learn from data without explicit programming, and deep learning is a subset of machine learning that makes use of multiple-layered neural networks to learn complex patterns from data.^195^ These techniques can denoise signals and extract spectral features, thereby enhancing the sensitivity and specificity of optical sensors.^196^^,^^197^ On the contrary, the models have since demonstrated rapid progress in automating the interpretation of image data from lateral-flow assays and microscopy, showing the feasibility of integrating AI-driven decision-making in resource-limited health systems.^197^ The AI-assisted optical decision-making can be divided into two approaches. The first approach includes a combination of feature extraction with classical ML methods, where features derived from data, such as images or spectra, are processed by the classical ML algorithms to predict concentrations. The second approach utilizes end-to-end deep learning architectures to infer diagnostic outcomes, as illustrated in Fig. 2.^197^

**Fig. 2:**
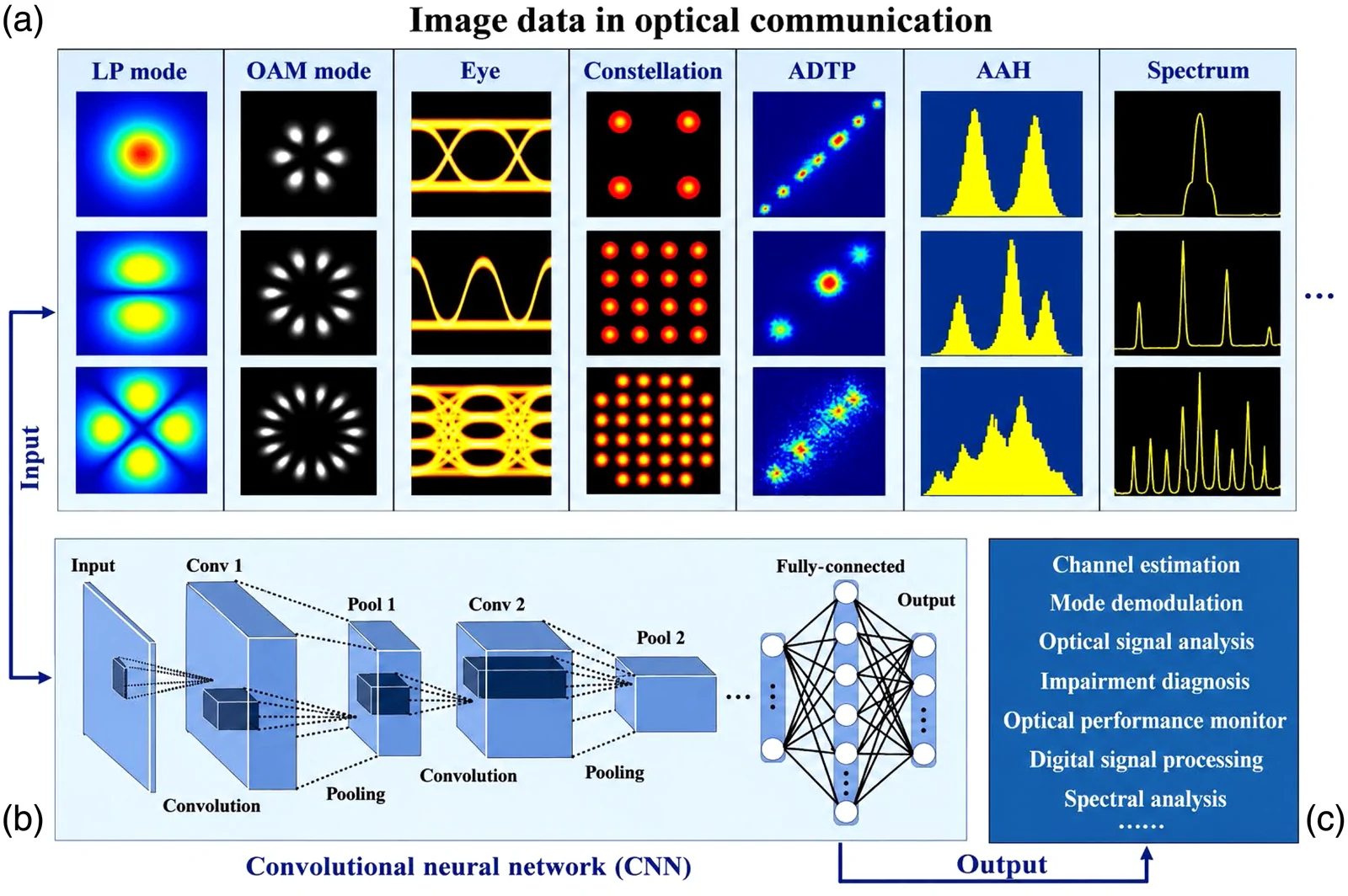
Workflow for generating output from optical data using a deep learning model.^197^

Although limited datasets have been identified as a major challenge to realizing AI applications not only in interpreting optical signals but in general, researchers have employed transfer learning to compensate for the limitations of optical signals, enabling the models to generalize the datasets.^197^ In addition, recent approaches explore the fusion of multimodal signals for optical signals to enhance data analysis and model performance.^197^ Empirical studies have demonstrated that these approaches have the potential to lower the limits of detection and reduce false negatives, thereby enabling the automated interpretation of optical signals. Although AI-assisted interpretations of optical signals are promising for improving decision-making accuracy, their successful implementation still requires careful consideration of training data practices. The training data should be diverse and free from bias to enable the models to generalize well across various clinical conditions.^196^

### 6.2 Edge Computing and Smartphone-Based Data Analysis

Edge computing is a distributed computing model that processes data close to its sources, such as Internet of Things (IoT) devices or smartphones.^198^^–^^200^ This offers advanced solutions to address limited bandwidth, constraints, latency, and privacy concerns that are particularly prevalent in resource-limited settings.^198^^,^^201^ In addition, by allowing preprocessing, encryption, and AI inference on a local server, edge computing can reduce the dependency on cloud connectivity and largely minimize the transfer of raw data.^198^ That is, edge-enabled applications eliminate possibilities of unauthorized access and data breaches. This is particularly suitable for a decentralized health system where real-time diagnosis is critical. The architecture of edge computing enables edge nodes to actively respond to service demands when integrated with IoT, providing efficient services to end-users.^202^ The edge computing functions in two primary ways: (1) it processes downstream data for cloud services and (2) handles upstream data for IoT services.^198^ In healthcare, these functions can assist in predicting, detecting, and preventing health challenges by deploying AI algorithms across edge devices that support low latency, local awareness, and low latency. The main characteristics of edge computing are outlined in Fig. 3.

**Fig. 3:**
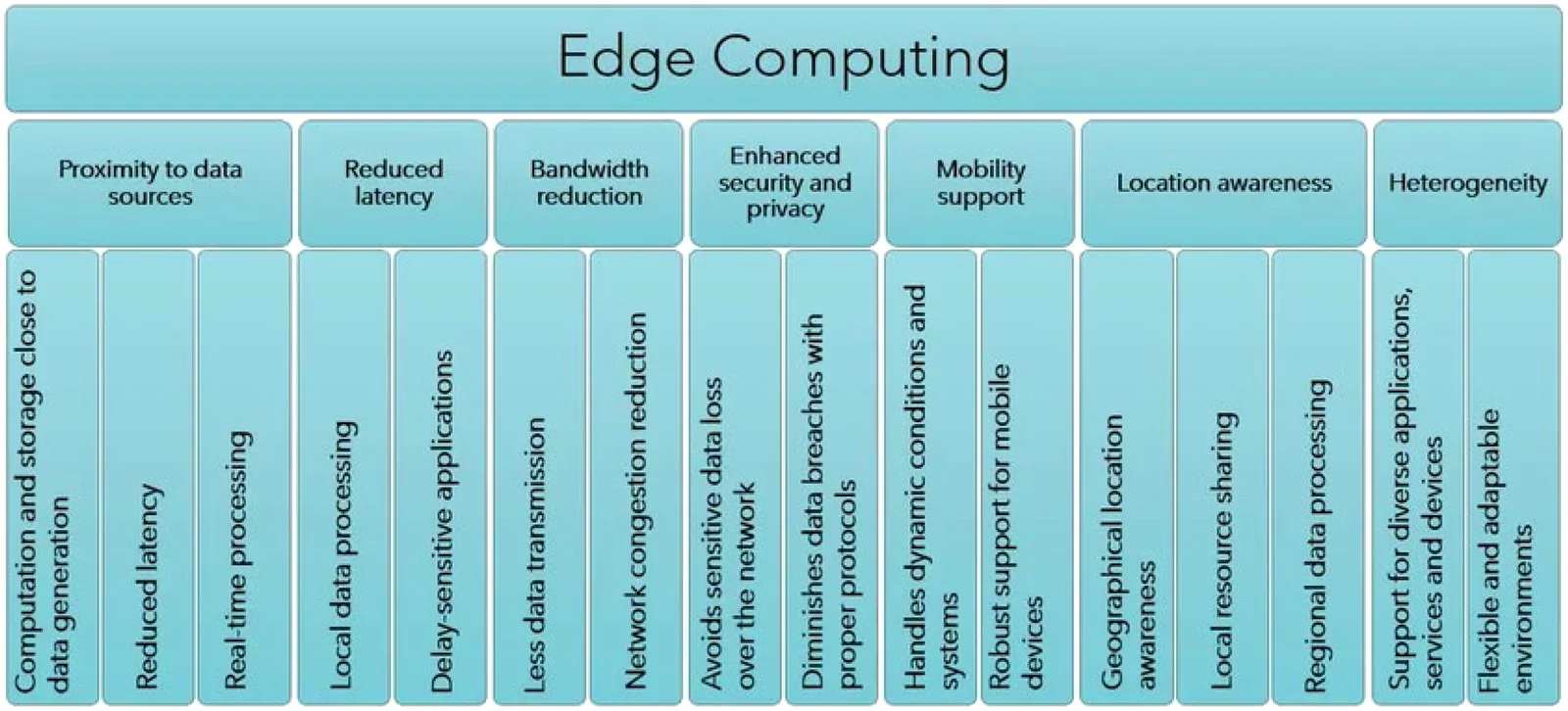
Main characteristics of edge computing.^198^

Several edge computing architectural strategies are also emerging to facilitate data analysis. Some include on-device inference, which permits immediate diagnostic feedback on smartphones through the central processing unit (CPU). The edge aggregation node, which provides intermediate privacy boundaries on data collected from multiple devices, then performs analyses before transmitting an encrypted summary to a cloud. Finally, split-computing models, which distribute layers of computation between edge devices and local servers.^198^ Although these strategies offer numerous advantages, such as reduced operational costs, they also present multiple challenges. The heterogeneity of both edge devices and smartphones necessitates progressive degradation strategies, whereas security measures should be applied. That is, updating an AI model across a widely distributed network device requires secure over-the-air (OTA) updates with robust logging for traceability.^198^ It is these related challenges that current research is prioritizing the development of standardized tool-chains for model compression that can maintain clinical calibration, as well as conducting comparative field studies to evaluate the cost-effectiveness and health outcomes of edge and cloud inference in rural areas and clinics.^198^

### 6.3 Cloud-Based Telemedicine and Health Informatics in South Africa

Cloud-based telemedicine and health informatics refer to the use of cloud computing technologies to deliver health care services and manage information remotely. In the context of South Africa, these technologies enable healthcare professionals to provide consultations, monitor patients, and analyze medical data through internet-based platforms. The adoption of telemedicine in South Africa was accelerated by the COVID-19 pandemic, as healthcare providers provided remote ways to deliver healthcare. This innovative shift has since been supported by the National Digital Health Strategies, which promote the use of digital platforms to enhance access, efficiency, and quality of healthcare services.^203^ Up-to-date South African healthcare institutions are increasingly deploying hybrid cloud infrastructures, integrating private clouds for sensitive data and public clouds for scalability and analytics, which enables the country to exhibit the highest level of telemedicine.^204^ This can be attributed to South Africa’s robust digital healthcare infrastructure and well-structured healthcare policies. The country has successfully integrated telemedicine tools, including AI-enabled chatbots, remote patient monitoring systems, and WhatsApp-based platforms. One of the successful telemedicine initiatives used in the country includes HelloDoctor, which offers mobile-based virtual consultations to enable patients to access medical advice remotely.^205^ This has improved access to healthcare for remote communities. As such, the Cloud-Based telemedicine and Health Informatics approaches in the country have proven to provide flexibility while maintaining compliance with national data protection laws.^206^ There are several factors that mainly contribute to South Africa’s high adoption of telemedicine, these include

• health provider readiness. The integration of the wearable medical devices and mobile health devices into the country’s healthcare ecosystem suggests high acceptance of telemedicine solutions.^204^^,^^207^
• digital infrastructure. The strong mobile and broadband networks in the urban areas of South Africa have enabled real-time teleconsultations and remote diagnostics.^208^^,^^209^
• government and institutional support. Existing South African policies and professional guidelines, such as the Health Professions Council of South Africa (HPCSA), provide a regulatory framework for health services in the country.^204^^,^^210^

However, connectivity remains a technical challenge, with uneven internet access affecting the reliability of cloud-based telemedicine services. This unevenness is reflected in the advancements within South Africa, as underserved communities experience limited access due to challenges such as affordability concerns.^208^ The country has then adopted telehealth applications with offline features to maintain service delivery in areas with limited connectivity.

### 6.4 Ethical, Privacy, and Data Governance Frameworks in African Health Systems

African health systems are increasingly adopting ethical, privacy, and data-governance frameworks to manage health data.^211^ Multiple countries have recently implemented data protection laws that vary across the countries. One of the laws is the Protection of Personal Information Act (POPIA), implemented in South Africa to govern how organisations handle personal information, is one of the comprehensive frameworks. Although policies have been established, they are not consistently applied in practice.^212^ That is, ethical concerns, particularly in health research, are primarily focused on informed consent, benefit-sharing, and engagement. However, the rise of big data has introduced new challenges, including data ownership, algorithmic bias, and secondary use. Nonetheless, identified gaps exist in integrating ethical principles into digital health policies, particularly in rural regions. As such, the adoption of privacy-abiding technologies is limited by factors such as low digital literacy and infrastructure limitations.^211^ Therefore, a multistakeholder approach, comprising governments, research institutions, health providers, and communities, would be crucial for co-developing ethical and data governance systems.^211^

### 6.5 Synthesis: Integration Pathway and Research Agenda

The synthesis of data analytics and digital health in the African health system requires a context-aware approach. This can be achieved by developing an interoperability framework that unifies fragmented health data sources into analysable systems. This has the potential to enable real-time surveillance, personalized care delivery, and predictive modeling. In addition, capacity building can be essential, particularly in digital literacy and data science, to empower local health workers and researchers to interpret data insights. Furthermore, government structures can be established to ensure the ethical use of data, privacy, and protection. On the contrary, the research agenda can pay close attention to evaluating the impacts of digital tools on health outcomes, identifying barriers to adopting these systems, and designing scalable models for rural settings. Priority should be given to AI-driven decision making (diagnostics) and telemedicine. There is also a need for cross-sector collaboration and adaptive policy frameworks to provide guidance for a suitable digital health integration.

## 7 Implementation, Policy, and Socioeconomic Perspectives

### 7.1 Technology Readiness and Translational Challenges

Despite significant advances in photonics and biophotonics that have yielded sensitive optical biosensors, miniaturized spectroscopy systems, and smartphone-integrated imaging platforms, the technology readiness of these innovations for widespread POC deployment in low-resource settings such as South Africa remains uneven. Many photonics-based prototypes achieve high analytical performance in controlled laboratory environments but have yet to attain the robustness, cost profile, and user simplicity required for real-world clinical integration. This gap reflects broader translational challenges documented across low- and middle-income settings, where technological sophistication often outpaces the readiness for implementation. In South Africa, stakeholder engagements have highlighted critical infrastructure constraints, including intermittent electricity due to persistent load shedding and limited digital connectivity, which impede the consistent use of digital-linked POC diagnostic models even where these tools exist.^6^ Moreover, healthcare workers’ capacity to adopt and maintain advanced optical devices is limited by training gaps, supply chain fragility, and insufficient quality management systems, a concern also raised in rural POC diagnostic studies emphasizing staff competency, supply continuity, and reliable performance monitoring.^5^ Regulatory pathways and evidence of clinical utility in target populations often lag technological development, delaying approvals and reimbursements that are essential for scale-up. Addressing these translational barriers will require integrated efforts across engineering, policy, and health systems strengthening to align photonics innovations with the ASSURED/REASSURED criteria for POC diagnostics and ensure that promising optical technologies advance beyond prototypes to sustainable tools that can meaningfully reduce diagnostic inequities in South Africa and similar contexts.^52^

### 7.2 Cost, Scalability, and Sustainability in African Contexts

The cost, scalability, and sustainability of photonics-based POC diagnostic technologies remain critical determinants of their impact in African health systems, where constrained budgets and vast rural populations amplify economic and logistical barriers. In many sub-Saharan settings, including South Africa, advanced POC platforms, particularly those integrating optical biosensing or miniaturized spectroscopy, entail significant upfront investment, with development costs for novel assays and devices reaching millions of dollars and influencing per-test pricing, which can deter adoption in low-resource clinical environments.^98^^,^^213^ Evidence from South Africa suggests that, although decentralized POC testing for HIV care can offer cost advantages in larger clinics, overall, per-patient costs for tests such as CD4 and creatinine at the POC may exceed those of centralized laboratory models, underscoring the dependence of cost-effectiveness on test volume and clinical context.^214^ Scalability is further hindered by limited local manufacturing capacity and supply chain fragility, often resulting in stock-outs and delayed delivery of consumables at rural clinics, a major sustainability challenge highlighted in stakeholder assessments of POC diagnostics across South African primary healthcare settings.^215^ Achieving equitable scale-up of photonics-enabled diagnostics, therefore, requires strategies that reduce production and per-test costs, such as high-volume manufacturing, cost-sharing partnerships, and transparent tiered pricing; build resilient distribution and maintenance systems, and align with national health financing frameworks such as South Africa’s National Health Insurance, designed to decentralize and equitize access to essential diagnostic services.^215^ By addressing these economic and systemic constraints, photonics-based POC technologies can move beyond pilot implementation toward sustainable integration in health systems confronting persistent diagnostic inequity.

### 7.3 Health Policy Alignment with the South African National Health Insurance

The ongoing implementation of South Africa’s National Health Insurance (NHI) represents a pivotal health policy reform aimed at achieving universal health coverage (UHC) by ensuring equitable access to quality healthcare services for all residents, regardless of socio-economic status or geographic location, which inherently aligns with the goals of decentralized and near-patient diagnostics. The NHI Act, assented to in 2024, establishes a pooled single-payer system that strategically purchases health services, including diagnostic tests, from both public and private providers to eliminate out-of-pocket costs at the POC and promote equitable service delivery across primary, secondary, and tertiary levels.^216^ Central to this reform is the prioritization of accessible, high-quality diagnostics within re-engineered primary health care services, as evidenced by expert calls to scale up POC diagnostic availability to improve timely clinical decision-making, reduce inequities in rural and underserved areas, and enhance overall health system efficiency.^215^ Moreover, the NHI’s emphasis on health technology assessment (HTA) in defining benefit packages and resource allocation underscores a policy environment that could support evidence-based integration of innovative photonics-based POC technologies into standardized care protocols, contingent upon demonstration of clinical utility and cost-effectiveness.^217^ As South Africa transitions through phased NHI implementation, the alignment of biophotonics and photonics-enabled POC diagnostics with national health priorities, via accreditation frameworks, digital health strategy integration, and robust evidence generation, will be critical to realizing equitable diagnostic access and fulfilling the transformative potential of optical technologies within a unified health system.^218^

### 7.4 Capacity Building, Local Manufacturing, and Skills Development

Strengthening capacity building, local manufacturing, and skills development is crucial to translating photonics-based POC diagnostics from prototype to a sustainable impact on the health system within South Africa and across Africa. Local production of medical diagnostics not only reduces reliance on imports and enhances supply chain resilience but also cultivates a skilled workforce capable of innovating, producing, and maintaining high-performance optical diagnostic tools. Initiatives such as South Africa’s Medical Device and Diagnostic Innovation Cluster (MeDDIC) and the Technology Innovation Agency (TIA) exemplify national efforts to stimulate an integrated innovation ecosystem that supports product development, regulatory knowledge, and human capital growth within the medtech sector.^219^ These efforts align with regional calls for investment in local diagnostics manufacturing, which highlight the potential for job creation, technology transfer, and economic growth through enhanced African manufacturing capacity for diagnostic tests.^220^ Academic and research institutions, including transdisciplinary hubs such as the Centre for Development and Implementation of POC Diagnostics at the University of Pretoria, are bridging the gap between research and real-world implementation by engaging postgraduate researchers, industry partners, and policy stakeholders in the context-sensitive development and evaluation of REASSURED diagnostic technologies.^221^ However, overcoming persistent barriers, such as limited infrastructure for quality-assured manufacturing, scarcity of engineers and technicians with expertise in photonics and optical system integration, and fragmented regulatory and quality control frameworks, remains a priority to ensure that local capacity translates into scalable, high-quality products for underserved communities. Concerted investments in training programs, industry–university partnerships, and quality management systems will be crucial to building a domestic pipeline of skilled professionals and robust manufacturing platforms that sustain long-term innovation and health equity in South Africa and beyond.

### 7.5 Case Studies of Successful Optical Diagnostic Deployments in South Africa

Emerging optical diagnostic technologies have begun to demonstrate real-world utility in South African clinical and research settings, underscoring the translational potential of photonics-based approaches to enhance healthcare delivery. At a tertiary eye care center in Johannesburg, OCT, a noninvasive, high-resolution imaging modality, has been successfully used to differentiate ocular surface squamous neoplasia from benign lesions with a sensitivity of 87.2% and specificity of 75.6% relative to histopathology, illustrating the value of optical imaging in augmenting clinical decision-making where histological services may be limited or delayed.^222^ In parallel, research efforts supported by local institutions such as the CSIR and the University of KwaZulu-Natal have developed and evaluated photonic crystal-based and surface-plasmon-enhanced optical biosensors tailored for TB detection, a high-burden disease in South Africa, with custom optical setups demonstrating the ability to monitor biomolecular interactions between mycobacterial antigens and antibodies in biosensing formats amenable to miniaturization.^223^ This review referenced prospective case-control study conducted on 175 patients, in whom 182 conjunctival masses were evaluated. The diagnostic performance of OCT (sensitivity 87.2% and specificity 75.6%) was assessed by comparing OCT findings with histopathology in 182 lesions, comprising 135 histologically confirmed OSSN and 47 benign lesions. This not only exemplify how optical coherence and biosensing technologies can be adapted for POC and near-patient diagnostics in South African contexts but also highlight productive intersections between engineering innovation and pressing public health needs. As these technologies advance toward field-ready prototypes and clinical evaluation pathways, they offer tangible models for how biophotonics can reduce diagnostic delays and improve access to high-quality care in resource-constrained environments.

## 8 Emerging Frontiers in Biophotonics for African Health Innovation

Biophotonics presents groundbreaking opportunities for research and industry, particularly in medicine, pharmaceuticals, food, and biotechnology. Innovative optical techniques allow scientists to capture cellular conditions and dynamic processes, offering a comprehensive view of life at the molecular to organ levels. By examining the ways in which light interacts with biological materials through absorption, emission, reflection, and scattering, these methods evaluate the structural, functional, mechanical, and chemical characteristics of those materials.^224^ Lasers and other cutting-edge light sources are also used in surgery and medical procedures. These innovations could make agriculture better, food monitoring, the environment, and medicine in addition to being useful in these fields.^224^

### 8.1 Hybrid Photonic–electronic and Microfluidic Platforms

The food sector, pharmaceuticals, oil and gas, environmental monitoring, and biomedical engineering are just a few of the sectors that use electrochemical sensors.^225^^–^^228^ These sensors promise to identify diseases and harmful items more quickly and sensitively.^229^^,^^230^ Similar to electronic integration, photonic integration aims to combine several optical operations into a small, dependable device with minimal power consumption. These days, there is not much convergence of photonic devices on one major material system. Most light sources are made of materials linked to III-V semiconductors. Microfluidics is the study of microstructures that can manage small fluid volumes. Numerous microfluidic architectures have been successfully utilized in lab-on-a-chip systems for a variety of fluidic processes.^231^^–^^236^ Microstructures known as microfluidic channels contain the fluid and enable controlled movement. Because of their potential to reduce traditional laboratory operations to a chip-level system, microfluidic devices have garnered a lot of interest.^237^^,^^238^

These apparatuses consist of closed microfluidic channels, chambers, and parts for a range of small-scale techniques and examinations. Blood nucleic acids and plasma and tear glucose levels can be quantitatively detected using portable microfluidic devices.^239^^,^^240^ A key element of precision medicine is POC diagnostics, which could become more reasonably priced as a result. Heart tissue models and lung functions can be replicated on a chip using biomimetic microfluidic devices.^241^ This might be used for drug testing, understanding the origins of disease, and finding new remedies using physiologically appropriate 3D organ-on-a-chip models. These miniature devices have the following benefits: low cost, quick reaction times, tiny reagent volumes, disposability, and decreased or eliminated cross-contamination.^242^

### 8.2 Nanophotonic and Quantum-Enhanced Sensing Approaches

The increasing demand for improved healthcare outcomes has driven substantial research into high-performance sensing technologies that can enable early disease detection. Among these, on-chip nanophotonic sensors have emerged as a promising class of devices because of their ability to offer compact, high-precision detection systems. Recent progress in integrated nanophotonic optical sensors has yielded remarkable improvements in sensitivity and detection limits, with reported values reaching up to 1000 nm per refractive index unit (RIU) and detection thresholds as low as picograms per millilitre (pg/mL).^10^

**On-chip nanophotonic sensors.** Photons are used as information carriers in on-chip topological nanophotonic devices, which offer strong topological protection while light is propagating. These gadgets have a lot of promise for photonic chips of the future. Operating at the nanoscale, on-chip nanophotonic systems process information with low energy consumption and broad bandwidth, making them attractive for applications such as all-optical interconnects, computing, and networking.^243^ These technologies are developed using a variety of fields, such as waveguide engineering, silicon photonics, guided-wave optics, nanophotonics, and plasmonics. In sophisticated functional materials such as semiconductors,^244^ phase-change materials,^245^ noble metals,^246^^,^^247^ and two-dimensional materials, subwavelength light–matter interactions combine classical and quantum effects at this scale.^243^^–^^248^ These engineered metamaterials, artificial materials with subwavelength features, enable unprecedented manipulation of optical properties. When incorporated as waveguide overlayers, metamaterials act as nanoscale anisotropic scatterers capable of tailoring the phase, amplitude, and polarization of light. This allows tuning of waveguide performance, including reducing facet reflection losses,^249^^,^^250^ engineering effective refractive indices to modify propagating modes,^251^ and even cloaking objects on chips.
**Metal-plasmonic nanophotonic sensors.** Plasmonic metal nanostructures have garnered extensive attention as optical platforms for sensing because of their capacity to strongly enhance fields and confine light at deep subwavelength scales via surface plasmon polaritons and localized surface plasmon resonances (SPPs).^252^ Surface plasmon resonance (SPR) biosensors are now considered the gold standard for label-free, instantaneous interaction among biomolecule analyses. Meta-surfaces applied to plasmonic waveguides can produce sophisticated effects such as invisibility cloaking. For example, Galutin et al.^253^ demonstrated that a meta-surface overlayer can suppress scattering from an object placed within a waveguide’s evanescent field, effectively rendering it invisible. High-dielectric spacers, such as silicon nano-spacers, enhance light confinement and facilitate coupling to hybrid plasmonic modes by engineering effective permittivity. In addition, meta-surfaces engraved in silicon waveguides have been shown to enable controlled mode conversion.^254^
**Quantum-enhanced sensing.** A core goal of quantum photonic sensing is to measure delicate samples using extremely weak optical signals without causing damage. By exciting surface plasmon polaritons, nanoscale confinement of electromagnetic near fields has produced notable advancements in quantum plasmonic sensors.^255^ These plasmonic modes tightly localize light past the limit of diffraction, dramatically improving interactions between light and matter additionally, amplifying otherwise undetectable optical signals. Consequently, quantum plasmonic devices demonstrate exceptional sensitivity, enabling applications such as single-molecule detection, real-time monitoring of biochemical reactions, and precise probing of changes in the local dielectric environment.^256^ These capabilities have positioned plasmon-enhanced quantum sensing as a leading approach in biomedical diagnostics, environmental monitoring, and nanoscale materials analysis.^257^
  Quantum biosensing (QB) offers broad potential, from tracking therapeutic molecules within cells to delineating tumor margins during treatment.^258^ Its high sensitivity may allow for the detection of single ions crossing membranes, the monitoring of neuronal synaptic activity, and the visualization of intracellular peptide transport, tasks that are nearly impossible with conventional imaging systems.^259^ The convergence of biology, sensing technologies, and quantum engineering stands to revolutionize medicine by providing molecular- and cellular-scale precision.^260^ Quantum biosensors, built using solid-state devices or free-space atoms and molecules, often rely on photon-based measurements to generate highly refined statistical data that enhance diagnostic accuracy.^261^ In global health, biosensors already play an important part in detecting infectious diseases, identifying drug-resistant organisms, and monitoring toxins and microbial contaminants. Quantum-enabled biosensing represents the next major leap in diagnostic performance.^262^
**Quantum biosensors for cell imaging.** Early disease detection drastically improves treatment outcomes. Modern biosensors offer high sensitivity, accuracy, rapid detection, and affordability. Emerging nanobiosensors further expand these capabilities by integrating nanomaterials with biological elements such as aptamers, nucleic acids, antigens, and receptors.^263^ By employing a 100-nm layer of dense NV centers, they achieved highly detailed magnetic imaging of tumor tissue labeled with magnetic nanoparticles, highlighting the strong potential of NV-based sensors for biological imaging.
**Quantum biosensors as wearable devices.** Wearable biosensors have attracted a lot of attention because they offer nonintrusive, instantaneous monitoring of biomarkers in fluids such as interstitial fluid, perspiration, tears, and saliva. Innovations in optical and electrochemical wearable biosensors have enabled measurement of hormones, pathogens, and metabolites with increasing precision and comfort.^264^^,^^265^ Modern wearable systems often incorporate microfluidics, multiplexed sensing, and efficient sample transport, enabling flexible and compact designs that are suitable for continuous health monitoring. Because the skin is an easily accessible organ rich in diagnostic biofluids, wearable quantum biosensors offer powerful opportunities for disease detection and health assessment.
**Quantum biosensors for disease diagnostics and drug monitoring.** Heart disease remains the leading cause of death worldwide, highlighting the need for quick and accurate diagnostic methods. Recent progress includes nanomaterial-based POC biosensors for detecting lipids and cardiac biomarkers such as LDL, HDL, and troponin I.^266^ Quantum dots (QDs) have strengthened these platforms through their bright, stable fluorescence, and strong signal amplification. Similar advancements have been reported in cancer diagnostics, where biosensors convert biomolecular signatures such as DNA, RNA, proteins, and exosomes into quantifiable optical or electrochemical outputs for early detection.^264^ Because of their high specificity and customizable emission, QDs are particularly useful in fluorescence-based genetic biosensors that detect microRNAs and single-nucleotide variations.^265^ By increasing surface area and being compatible with cutting-edge methods such as SERS, other nanomaterials including carbon nanotubes, graphene oxide, and gold nanoparticles increase sensitivity.^267^^,^^268^ When combined, these technologies provide effective instruments for treatment monitoring and early disease diagnosis.
**Challenges and future directions for quantum biosensors.** Despite rapid scientific advances, significant gaps remain between laboratory successes and the commercialization of quantum biosensing (QB) technologies. Reducing nonspecific binding, decreasing background noise, and attaining high sensitivity and specificity in intricate biological contexts are major challenges. Overcoming these challenges will require improved surface functionalization, advanced signal processing, and the integration of machine-learning tools.^269^ Commercialization also demands compact, portable devices, yet miniaturization often compromises performance. Innovations in nanostructuring, microfabrication, and microfluidics will be essential for enabling precise sample handling without sacrificing detection sensitivity.
  Additional barriers to wider use include scalability, high production costs, and material constraints. Modern coatings, surface treatments, and encapsulation techniques will be needed to increase stability and biocompatibility over the long term while halting degradation in biological media. Successful translation of QB technologies will rely on strong collaboration among researchers, industry partners, and regulatory bodies, supported by clinical validation to ensure safety and reliability. Additional challenges include simplifying device operation, ensuring clinical acceptance, and enabling multiplexed biomarker analysis. Addressing these issues is crucial to meeting the growing need for affordable, high-sensitivity diagnostic platforms that deliver rapid, reliable results with low system complexity.

### 8.3 AI-Driven Adaptive Diagnostics and Digital Twins

The use of AI in healthcare is steadily increasing and rapidly changing how medical diagnosis and treatment are delivered. AI-based diagnostic solutions are made to simulate how the human brain processes enormous volumes of data for diagnosis. From analytical models that assist in patient analysis and outcome prediction to imaging and analysis technologies that assist radiologists in interpreting pictures. When compared with human professionals, AI-based solutions may not only help with diagnosis but also greatly improve therapy outcomes and lower errors, making them a crucial tool for creating tailored medical plans.^270^^,^^271^

Artificial intelligence is progressively making its way into patient prognosis, image-based diagnosis, and supporting physicians in clinical decision-making.^270^ High-income countries with advanced healthcare systems that are well-developed, well-developed, and restructured with effective technological systems have adopted AI rapidly. For instance, in the USA, AI technologies are now essential to software applications, management, patient precision medicine, and diagnostic imaging and reporting in pathology.^272^ Not every nation on the planet has fully embraced AI. There are many obstacles to the adoption of AI technology in low- and middle-income nations, such as exorbitant costs, insufficient infrastructure, and a shortage of skilled workers.^273^ Moreover, these adoption differences are limited to a certain kind of healthcare facility rather than a particular geographic area. Larger organizations, including public and private hospitals, are also seen to adopt AI technologies more swiftly since they have more resources and employ specialist human resources.^274^

AI can improve diagnoses by consistently reaching diagnostic qualities that are superior to those of human doctors, as shown by Esteva et al.^271^ McKinney et al.^275^ have shown, for example, that AI algorithms can diagnose some cancers from imaging scans more accurately than radiologists. They may also be able to delay diagnostic errors, which are the main cause of patient injury in all medical facilities worldwide.^276^ In addition to accuracy, AIDTs can improve healthcare systems. AI can save time in treatment planning, just as it can in diagnosis, and several healthcare professionals facilitates the provision of real attention to cases that need it.^277^ This effectiveness has the potential to reduce expenses and boost patient volume, which is an important consideration in a context with limited resources, as indicated in Table 2 below.^271^ Personalized medicine, a crucial field where AI techniques are particularly helpful, is the growth of the customized approach in healthcare. By processing datasets that contain genetic information, lifestyle characteristics, and clinical history, AI may help medical professionals predict precise treatments with the potential to improve clinical results and reduce side effects.^278^

**Table 2:** Comparison between AI-based and conventional methods for diagnosing diseases.

Aspect	Conventional methods	AI-based disease methods
Accuracy	Varies by clinician expertise; risk of human error	Often higher due to data-driven analysis and pattern recognition
Speed	Slower; depends on manual evaluation	Very fast; can process large datasets in seconds
Stability	Consistency may vary among clinicians	Highly consistent, same input yields same output
Cost	Can be high due to consultations, tests, and labor	Initially high setup but low per-use cost; scalable
Subjectivity	Influenced by clinician judgment and experience	Minimal subjectivity; relies on objective data
Early detection	Limited by human ability to notice subtle signs	Strong ability to detect early or hidden patterns
Personalization	Generalized approaches; personalization depends on the clinician.	Highly personalized insights based on individual data
Complex pattern recognition	Difficult for humans to detect complex, high-dimensional patterns	Excellent at recognizing complex, nonlinear patterns
Error detection	Hard to detect rare or overlooked errors	Can flag anomalies, inconsistencies, and rare findings
Data utilization	Limited ability to process large datasets	Capable of using massive amounts of structured and unstructured data

The use of Digital Twins (DTs) is one viable remedy in this respect. A digital representation of a real-world system, item, or someone that is continuously updated with sensor data is called a DT. DTs serve as virtual patient representations in the healthcare industry by combining clinical, demographic, and biometric data to provide comprehensive patient profiles.^279^ Healthcare practitioners can more accurately predict treatment outcomes and customize interventions using DTs to simulate and analyze patient-specific situations. In the African healthcare context, DTs hold significant potential for advancing personalized and predictive medicine, enhancing operational efficiency within hospitals, and strengthening telemedicine and remote monitoring capabilities.^279^ They support simulations of treatment plans, provide real-time insights into patient health, and optimize hospital resource allocation. In addition, DTs contribute to public health initiatives and serve as valuable tools for training healthcare practitioners.

In addition, DTs are essential in lowering medical procedure errors and unfavorable patient management results. One prominent use is in the Safety 4.0 framework, which is an advancement in workplace safety influenced by Industry 4.0 technology. Safety 4.0 leverages big data, artificial intelligence, and augmented reality to improve safety practices and promote more efficient, secure working environments. When integrated into this framework, DTs greatly lower human error and assist in more accurately managing intricate safety procedures.^280^ Vaccine program development, vaccine supply chain optimization, acceptance rate prediction, and the identification of vaccination barriers are all significantly impacted by them. Healthcare organizations frequently encounter challenges, including an excessive amount of patient data, privacy issues, and restrictions on data transmission. Clinical research and medicine have been revolutionized by DT technology, which allows users to ask insightful questions, obtain better responses, and utilize data to get useful insights while protecting the privacy and health of actual people.^281^ This technology makes it possible to organize information and knowledge effectively, which improves patient care, research outputs, and decision-making.

The field of geriatric medicine has received wide application of DT as it has been used to facilitate personalized care, remote health data tracking, and proactive patient involvement.^282^ DTs improve response times and actions by facilitating early data collection and anomaly detection.^281^ As demonstrated during the COVID-19 pandemic, they managed critical care resources and simulated changes in workflow to maximize hospital efficiency.^283^ This extensive use of DTs in healthcare is a prime example of how they have revolutionized both patient care and healthcare administration.^284^ Drug discovery and biomanufacturing have also made use of DTs. Efficiency has become embedded in the pharmaceutical production process by reducing costs, schedules, and attrition rates associated with conventional procedures.^285^ The recognition and confirmation of viable therapeutic targets has been accelerated by scaling up the screening of ADME-Tox characteristics through the integration of DT modeling with computer- assisted medication development and machine learning approaches. Drugs for illnesses including HIV and cancer have been introduced as a result of this technique.^286^ By lowering errors and enhancing results, DT technology has improved surgical planning through the use of accurate, patient-specific anatomical models. For instance, trans-catheter aortic valve replacement (TAVR) is one of the complicated cardiac procedures that cardiac surgeons can do using DT models such as Heart Navigator.^287^^,^^288^

### 8.4 Sustainable and Circular Design for Low-Resource Manufacturing

Circular economy (CE) models that incorporate AI have the potential to completely transform industry-wide sustainability initiatives. Prioritizing context-sensitive, robust, and repairable designs that overcome data and infrastructure limitations while employing AI for resource optimization is essential for sustainable and circular design for low-resource manufacturing of digital technologies (DT) and artificial intelligence in healthcare. In settings with limited financial resources and technical expertise, the fundamental approach entails incorporating circular economy (CE) concepts throughout the whole product lifespan, from design to end-of-life management.

Manufacturing photonic components relies heavily on cleanrooms, wafer-processing steps, and chemical etching, each of which carries a substantial environmental footprint. Life-cycle assessments (LCAs) have become essential for quantifying these impacts. In photovoltaics, one of the largest and most mature areas of photonics, the polysilicon supply chain achieves a Material Circularity Indicator of only 0.54, reflecting limited material recovery and recycling efficiency.^289^ Projections further indicate that by 2050, silicon PV modules may still emit between 8 and 13 g ${\mathrm{CO}}_{2}$-equivalent per kilowatt-hour of electricity produced,^290^ suggesting that even as electrical grids decarbonize, the energy and carbon embedded in manufacturing will remain comparatively high.

Cleanroom operation represents one of the most significant contributors to this footprint. These facilities run continuously to maintain stringent particle filtration, controlled airflow, and stable temperature and humidity levels; conditions essential for high-yield photonic fabrication. The tools within them also operate for extended periods because disruptions increase the risk of contamination. Collectively, these requirements make semiconductor and photonics cleanrooms among the most energy-intensive environments in the technology manufacturing sector.

Chemical processing further amplifies the environmental challenges. Plasma etching, for example, relies on fluorinated greenhouse gases with global warming potential orders of magnitude higher than ${\mathrm{CO}}_{2}$.^291^ In parallel, photonic manufacturing draws heavily on ultrapure water and rare-earth elements, placing additional strain on natural resources. Despite their importance, detailed datasets describing these chemical and resource flows remain sparse, leaving parts of the environmental footprint insufficiently quantified and, at times, easily overlooked.

### 8.5 Future Roadmap for Biophotonics in South African Health Innovation

The creation of POC diagnostic instruments for illnesses such as COVID-19, TB, and HIV as well as drug delivery and discovery systems utilizing photonics-based methods, are the primary objectives of the future roadmap for biophotonics in South African health innovation. Scientists can observe and identify diseases, such as HIV transmission, using biophotonics. Due to the potential for employing UV light rays to treat such disorders, medical professionals worldwide have become increasingly interested in biophotonics therapy (BPT). By exposing blood to UV radiation, BPT stimulates the immune system to eliminate all infections, including bacterial, fungal, and viral ones.^292^ BPT is also known as photoluminescence, photopheresis, photodynamic therapy, hemotoxic oxidative therapy, and UV blood irradiation (UBI). Typhoid, wound infections, pneumonia, septicemia, and peritonitis are among the bacterial infections that BPT has been shown to be quite successful in treating.

Recent South African research as reported by Mthunzi-Kufa,^293^ who presented their study results on label-free, laser-driven methods for identifying HIV-1, the most common form globally, in biological cells that are alive, illuminates some of the significant work in the use of biophotonics in HIV/AIDS diagnosis and treatment. Laser technology is hardly used as a technological instrument to research HIV-infected cells, which are also used for diagnosis, even though it is used to detect and treat cancer cells. Lugongolo et al.^294^ have focused on the identification of HIV-1-infected and uninfected cells utilizing Raman and transmission spectroscopy in combination with optical trapping. Combining laser trapping with Raman spectroscopy allows for the first time in HIV research to immobilize and analyze single infected cells without the need for labels, in contrast to the gold-standard HIV-1 diagnostics of the enzyme-linked immunosorbent assay and nucleic acid-based tests, which both require the use of labels and substrates. To diagnose diseases such as HIV/AIDS, the optical system basically enables the capture of specific cells whenever desired, and the analysis of their chemical fingerprint by merely illuminating them using laser light of various wavelengths and elaborately designed beams.^295^

Reverse transcription quantitative polymerase chain reaction (RT-QPCR) and flow cytometry are typical sensitive techniques for measuring viral load and CD4 cell count to track ART, but they need expensive chemicals, sophisticated lab equipment, and trained personnel.^296^ SPR and LSPR are two examples of the optical, electrical, and acoustic sensing techniques used in a number of virus detection systems. Using gold nanoparticles that self-assemble and are labeled with biotinylated anti-gp120 antibodies, as determined using viral lysate samples and impedance spectroscopy, a platform based on nanoplasmonics was developed to identify intact HIV-1.^297^ Among these strategies, biosensors using photonic crystals (PC), which track variations in dielectric permittivity at the interface between a transducer substrate and liquid media, provide a quick and accurate optical detection technique for biomolecules, cells, and viruses.^298^ In addition, a PC biosensing platform was created that can identify and detect biomolecules, antibodies, and whole viruses (HIV-1).

The roadmap includes leveraging technologies such as super-resolution imaging, spectroscopic techniques, and biosensors to facilitate single-molecule and cellular studies. A unique, noninvasive, and genuinely helpful treatment for a number of diseases, including cancer and noncancerous ones, is photodynamic therapy (PDT).^299^^–^^303^ PDT is a treatment in which a photosensitizer is activated by a particular kind of light. To kill cancer cells, the photosensitizer absorbs light and creates a hazardous form of oxygen. Furthermore, through blood vessel rupture, deprivation of nutrients necessary for survival, and stimulation of the immune system to target the cancer cells, PDT destroys cancer cells. New strategies for PDT penetration have been made possible by the development of bionanotechnology. PDT aims to target and kill particular cell types using light and a light-sensitive drug called a photosensitizer.^304^^–^^306^ As a treatment for cancer and other diseases, PDT mainly targets surface malignancies.^307^^–^^311^ Several studies on biophotonics projects are underway in South Africa, including low-level laser therapy, optical trapping, and spectroscopy for HIV infection detection, Raman spectroscopy for disease detection and drug screening, and surface plasmon resonance for quantitative studies, to name a few. By developing effective, laser-based POC instruments for better healthcare services, these researchers hope to improve the lives of South Africans, especially those living in environments with little resources.^312^^,^^313^

The future roadmap involves integrating biophotonics with ML and AI to enable smart diagnosis, real-time health monitoring, and personalized treatment planning. Important organizations, such as the Council for Scientific and Industrial Research (CSIR) and other biophotonics investigation groups, are central to this roadmap, housing the National Photonics Centre to equip industries with the necessary tools and processes. The goal is to mature technologies to a high readiness level for commercial transfer and to support local bio-based technology growth within the national bioeconomy strategy. Advancing biophotonics requires sustained investment from government agencies, the private sector, and public-private partnerships. National strategies, such as the South African Research Infrastructure Roadmap and the Bioeconomy Strategy, which promote bio-innovation for the advancement of society and the economy, serve as a basis for these initiatives. The production of vaccines and other health advances is among the Department of Science, Technology, and Innovation’s (DSTI) top goals for 2024 to 2025, which will create a favorable environment for associated biophotonics applications.

## 9 Conclusions and Outlook

South African case studies of optical and photonic-based diagnostic deployments demonstrate that a meaningful clinical impact is achievable when technologies are co-designed with end-users, embedded within existing health workflows, and supported by sustained training and maintenance structures. Implementations such as mobile retinal screening programs, LED fluorescence-based tuberculosis diagnostics, and Raman spectroscopy pilots for oncology services highlight a recurring theme: photonics succeeds in low-resource contexts when robustness, simplicity, and local adaptability outweigh the complexity of laboratory-grade systems. These cases collectively reveal a broader insight: technological excellence alone is insufficient without alignment to community needs, health-system constraints, and long-term operational feasibility.

The translational opportunities for biophotonics in resource-limited regions are substantial. The continent’s high burden of infectious and noncommunicable diseases, combined with limited access to advanced laboratory infrastructure, positions optical techniques as a uniquely enabling platform. Their inherent sensitivity, low-power requirements, and potential for miniaturization support development of POC formats that circumvent conventional bottlenecks in diagnostics. In South Africa, convergence between photonics innovation hubs, engineering groups, and clinical partners is accelerating the maturation of emerging technologies from integrated photonic chips to low-cost spectroscopy, optical biosensors, and AI-augmented image-based diagnostics. However, successful translation requires deliberate navigation of regulatory pathways, rigorous field validation, and integration with existing digital health systems. Addressing these factors early in the development pipeline may shorten the time between laboratory discovery and community-level impact.

Looking ahead, the vision for equitable and sustainable diagnostic futures in South Africa and similar low-resource settings is grounded in three key pillars: local manufacturing capacity, skills development, and health policy alignment. Expanding local fabrication capabilities for optical components and diagnostic consumables can reduce costs, ensure supply-chain resilience, and promote economic participation. Parallel investment in technician training, postgraduate programs and interdisciplinary photonics curricula will cultivate a workforce capable of maintaining, adapting, and innovating diagnostic systems. Finally, strategic alignment with the South African National Health Insurance (NHI) framework will ensure that photonics-based diagnostics are not merely technological demonstrations but integrated tools for universal health coverage, capable of addressing inequities in rural, peri-urban, and township environments.

Overall, the evidence suggests that photonics is poised to significantly transform the diagnostic landscape in Africa, if technology development is embedded within broader socio-technical systems that prioritize accessibility, long-term sustainability, and community trust. As research groups, clinicians, policymakers, and industry partners converge around shared translational goals, South Africa has the potential to serve as a continental leader in equitable optical diagnostic innovation. Continued investment in community-driven design, field-ready engineering, and systemic support mechanisms will shape a future in which advanced diagnostics are not a privilege of the few but a foundation for resilient and inclusive healthcare systems.

## Data Availability

Data and code sharing are not applicable to this study. No datasets or code were generated or analyzed that require separate access for replication or interpretation of the findings. The published article may be accessed and cited for scholarly and referencing purposes in accordance with the applicable license.
